# Emulsification Complexity of Silicone Oil in Retinal Surgery: In Vitro Insights into Phase Behavior

**DOI:** 10.1021/acsomega.5c09041

**Published:** 2026-01-09

**Authors:** Barbora Kamenická, Věra Pěnkavová, Eliška Lyko Vachková, Sandra Orvalho, Mária Zedníková, Natalie Jaklová, Petr Stavárek, Monika Reháčková, Patrik Rajs, Miroslav Veith, Petr Klusoň

**Affiliations:** † Institute of Chemical Process Fundamentals of the Czech Academy of Sciences, Rozvojová 135, Prague 165 00, Czech Republic; ‡ University of Chemistry and Technology Prague, Technická 5, Prague 166 28, Czech Republic; § Ophthalmology Clinic of the University Hospital Kralovske Vinohrady, Šrobárova 50, Prague 100 34, Czech Republic; ∥ Third Faculty of Medicine, 60571 Charles University, Ruská 87, Prague 100 00, Czech Republic; ⊥ Institute for Environmental Studies, Faculty of Science, Charles University in Prague, Benátská 2, Prague 110 00, Czech Republic

## Abstract

Silicone oil (SO) is widely used as an intraocular tamponade in retinal detachment surgery, but its postoperative emulsification is associated with several acute complications. This study investigates the physicochemical and interfacial mechanisms behind SO emulsification in vitro with a focus on biomolecular interactions at the oil–water interface. Using model aqueous humor containing albumin and γ-globulins, we observed the formation of both oil-in-water and water-in-oil emulsions depending on water/oil ratios. At a 5:5 water/oil ratio, oil droplets in the aqueous phase ranged 2.6–28.1 μm and aqueous droplets in oil 2.8–118.3 μm. At 1:9, only water-in-oil emulsions were formed (5–231.8 μm), and at 9:1, only oil-in-water emulsions occurred (≤5 μm). Moreover, protein-rich conditions significantly increased the emulsion stability and altered the interfacial rheology. Interfacial viscoelasticity measurements with SO 971 mPa·s indicated that proteins enhanced elasticity and interfacial structuring. Interestingly, the interface between model aqueous humor and SO 4990 mPa·s showed more dissipative behavior, despite this interface being a little more rigid in terms of complex modulus than SO 971 mPa·s. Inorganic salts or elevated temperatures had minor or negligible influence, while mechanical agitation was essential for emulsion formation. The formation of stable, small oil-in-water droplets highlights a potential pathway for their migration into the anterior chamber and through the trabecular meshwork, possibly contributing to chronic inflammatory and pressure-related complications. These results offer new insights into the interfacial behavior of SO in contact with biologically relevant fluids and underline the importance of understanding protein-mediated emulsification in the context of intraocular biomaterials.

## Graphical abstract

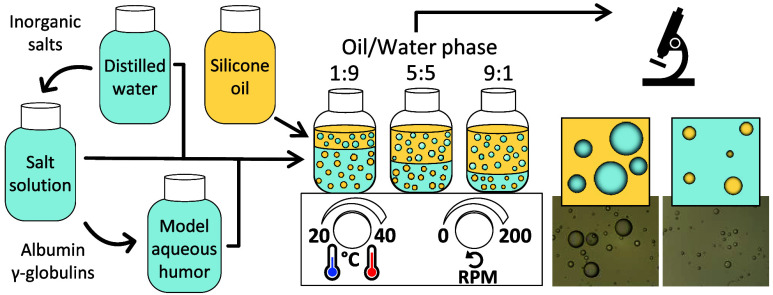

## 1 Introduction

In clinical practice, the separation of the neurosensory retina from the underlying retinal pigment epithelium is defined as retinal detachment. The two most common reattachment procedures performed today are scleral buckling and pars plana vitrectomy.^1^ In pars plana vitrectomy, vitreous humor is removed from the vitreous cavity to release vitreoretinal traction and an air–fluid exchange is usually performed to reattach the retina.^2^ Then, intraocular tamponades are utilized to maintain retinal break closure until the retinopexy matures to full strength.^1^

In last two decades, silicone oil (SO) has become an attractive substitute for the vitreous humor due to its high surface tension, ease of removal, low toxicity, and good transparency, which allows a clear view of the fundus.^3–5^ On the other hand, significant production of SO emulsions has a strictly undesirable side effect which plays a key role in complications such as optic neuropathy, acute and chronic changes in intraocular pressure (secondary glaucoma), extraocular migration, retinal and macular damage, corneal alterations, and cataract formation.^6^ Intraocular inflammation has also been previously reported in connection with SO emulsification.^7–10^

Recently, oil-in-water (O/W) emulsion has been widely described as a phenomenon in SO emulsification associated with retinal detachment.^3,6,11,12^ In reality, the breakup of oil into droplets and its dispersion in a thin layer of water near the retina may enable SO to migrate from the vitreous cavity to the anterior chamber and contact the cornea and trabeculum.^12,13^ SO droplets may also migrate into the subretinal space through a retinal break and obstruct the replacement of the detached retina.^3^ The oil droplets, which are transported to optical path, interfere with the vision.^3,6,11,12^ In addition to O/W droplets, Miller et al.^11^ mentioned that water-in-oil (W/O) emulsions are rare in the clinical setting.

Besides that, several factors that affect SO emulsification are discussed in the literature.^7,11,13–15^ Recently papers scrutinized the role of different SO properties, such as viscosity and molecular weight. For example, Miller et al.^11^ considered the eventuality that the SO with higher viscosity is more resistant to deformation and, therefore, it is less prone to disperse and eventually emulsify. Nevertheless, the simplicity of the injection method can be reached within a lower viscosity of SO and make this SO more attractive to some surgeons.^14^ Moreover, blood biomolecules presented in an aqueous phase can act as emulsifiers for SO, because they can absorb at the water/oil interface and modify its mechanical interfacial properties.^11,16^ The interface between SO and aqueous humor enriched with proteins such as albumin and γ-globulins represents a biologically relevant system, where protein absorption significantly alters interfacial viscoelasticity and promotes emulsion formation.^17^ From a theoretical perspective, the behavior of such soft interfaces is governed by a balance of interfacial tension, elastic modulus, and energy dissipation, all of which are influenced by the presence of surfactants or biomolecules.^18^ The research group of Nepita^6,19^ observed that the presence of albumin and γ-globulins in an aqueous solution had a significant effect on the water/oil interface, resulting in stable emulsions on the time scale of months. Other factors or parameters, such as interfacial tension^13^ or eye movements^6,14^ affecting the emulsification of intraocular SO, have also been reported.

However, the synergy of the mentioned factors is crucial to understanding the emulsification state and avoiding this phenomenon. Therefore, this study investigates the physicochemical and interfacial mechanisms underlying SO emulsification in biologically relevant environments. Emphasis is placed on the role of proteins in the aqueous phase, which absorb at the SO/water interface and significantly alter its viscoelastic properties. Systematic variation of water/oil ratios, temperature, and protein composition is compared in this article with the aim to reveal how biointerfaces govern emulsion morphology and longevity under intraocular-like conditions.

## 2 Materials and Methods

### 2.1 Materials

Commercial-grade SOs (poly­(dimethylsiloxane)) Brookfield Silicone Fluid 1000 cP (denoted as SO or SO1000) and Brookfield Silicone Fluid 5000 cP (denoted as SO5000) with viscosities of 971 and 4990 mPa.s at 25 °C, respectively, were obtained from AMETEK Brookfield (Middleboro, USA). Albumin and γ-globulins in ≥ 99% quality and form of lyophilized powder were purchased from Sigma-Aldrich (Prague, Czech Republic). Inorganic salts (NaCl, KCl, Na_2_SO_4_, NaHCO_3_, and CaCO_3_; p.a. grade) were acquired from Lach-Ner Co. (Neratovice, Czech Republic). Methylene Orange (MO; >99%, solubility in water 5 g L^–1^ at 20 °C; log Pow = −0.66; Sigma-Aldrich Prague, Czech Republic) was used for the dying of emulsions with the aim of distinguishing water and oil droplets.

The aqueous salt solution (SS) containing inorganic salts and model aqueous humor (MAH) containing the same salts in addition to human proteins, albumin, and γ-globulins were prepared. Inorganic salts and protein concentrations were taken from the literature^20,21^ and they are presented in Table 1. Distilled water (DW) was used to prepare all of the solutions.

**Table 1:** Composition of the Aqueous Phases Utilized in Emulsification Experiments^a^

component	concentration in SS (g/100 mL)	concentration in MAH (g/100 mL)
NaCl	0.7080	0.7080
KCl	0.0360	0.0360
NaHCO_3_	0.0020	0.0020
CaCO_3_	0.0020	0.0020
Na_2_SO_4_	0.0090	0.0090
albumin	0	0.0069
γ-globulins	0	0.0123

a Note: Concentrations based on refs 20 and 21 .

The porcine vitreous humor in satisfactory quality and the SO recovered from the vitreous cavity were supplied and evaluated by colleagues of the Ophthalmology Clinic of the University Hospital Královské Vinohrady, Prague. Vitreous humor is typically removed via a pars-plane vitrectomy procedure. Medical-grade SO was removed from the vitreous cavity during pars plana vitrectomy using special syringe equipment and a Constellation Vision system (Alcon, Fort Worth, TX, USA). The vacuum syringe with a blunt capillary needle was inserted into the cavity via a port, and the oil was aspirated. The syringe with the sample of SO was stored with a plastic cap until analysis.

### 2.2 Emulsification of SO

One of the aqueous phases (DW, SS, or MAH) and the SO1000 phase were placed in glass vials (inner dimensions: diameter 25 mm, height 40 mm; 20 mL in volume) in different weight ratios (1:9, 3:7, 5:5, 7:3, and 9:1) to a total weight of 10 g. The mixtures were shaken in circular motion at 200 rpm using a laboratory shaker GLF 3005 equipped with a vial holder. The rotary shaking of water/oil mixtures in vials was intended to mimic hydrodynamic conditions of the oil in the eye. All experiments were carried out at 20 and 40 °C. After 24 h of vigorous shaking, the suspensions were collected from the laboratory shaker. The water/oil mixtures were then kept in static conditions at 20 or 40 °C for 72 h. The samples of the oil and water phase were taken using a syringe with a needle (it must be mentioned that the O/W emulsions for SO/DW or SS ratios 9:1 and 7:3 could not be evaluated due to difficulty in sampling of the aqueous phase). The emulsions were subsequently analyzed via microscope image acquisition. Figure 1 presents the experimental protocol of the emulsification tests and Figure S1 in the Supporting Information presents all variants of the experiments performed.

**Figure 1:**
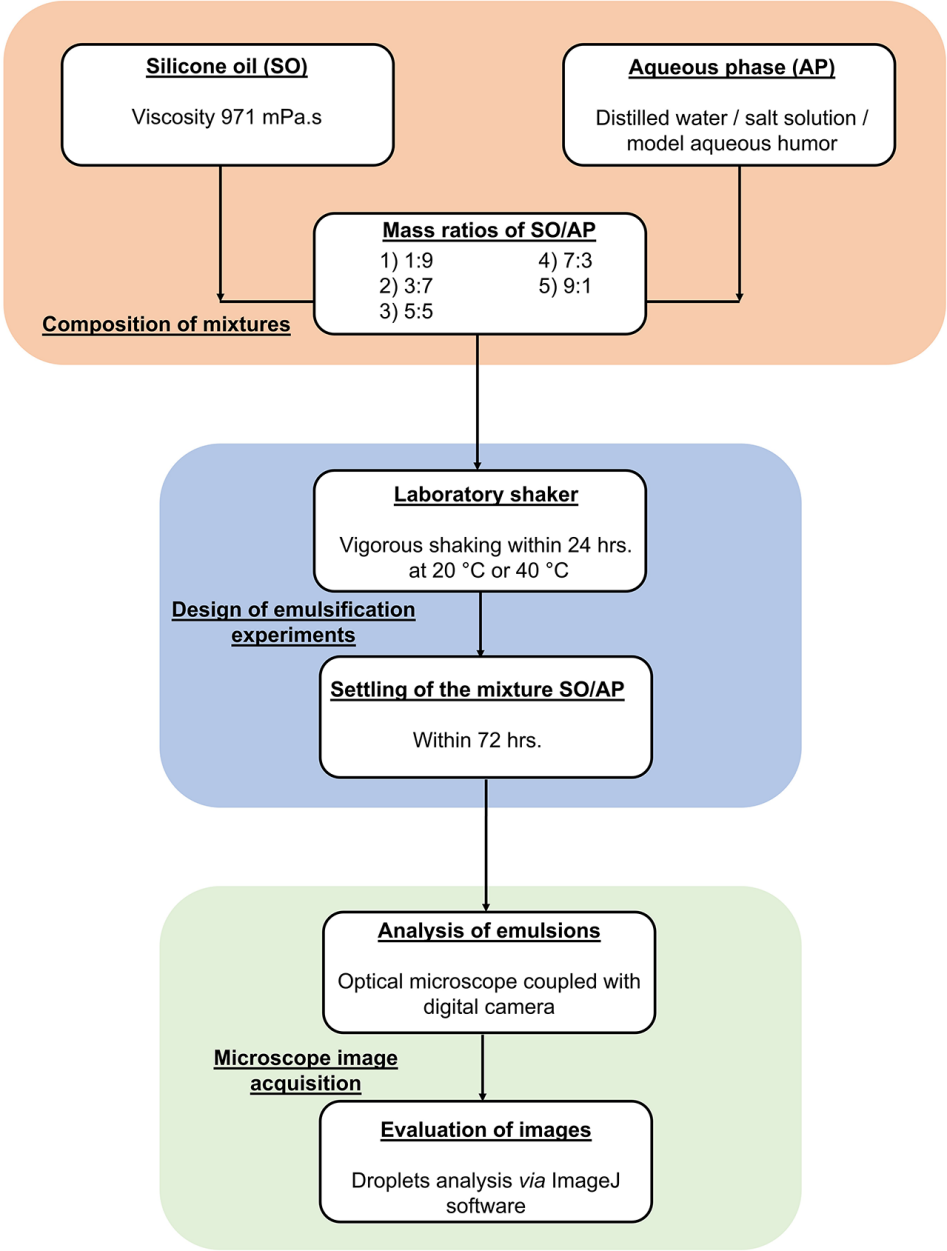
Experimental protocol of emulsification tests.

The stability of emulsions formed was continuously analyzed via the microscope image acquisition during the period of 4 months at static conditions. To observe the spontaneous emulsification phenomenon (emulsions are formed without mechanical agitation^22^), SO and DW or MAH were placed in weight ratio = 5:5 in a vial without shaking and kept at room temperature. After 1 month, the possible spontaneous emulsion was analyzed via microscope image acquisition.

### 2.3 Vitreous Humor Emulsification

To simulate the behavior of vitreous humor after vitrectomy, liquefied porcine vitreous humor (0.01 g) and 5 g of SO were placed in a round bottomed glass flask with a volume of 25 mL. After 2 h of vigorous shaking at 200 rpm, the flask was demounted from the laboratory shaker. Then, the mixture of liquefied porcine vitreous humor and oil was allowed to settle for 72 h and subsequently analyzed via the microscope image acquisition.

### 2.4 Microscope Image Acquisition and Droplet Size Evaluation

All of the formed emulsions were characterized with a microscope quantitative analysis. The samples of O/W or W/O were taken separately and identified on microscope slides. A ME-2665 optical microscope (Euromex GmbH, Duiven, Holland), equipped with objective (S.PlanM 10 × 0.25∞/0; Euromex, GmbH, Duiven, Holland) and digital camera (DSLR Nikon, D300s) installed on the microscope using the G-mount, was used to obtain images of the droplets. All the images were captured at 100× magnification and taken/processed using DigiCam Control software.

ImageJ software (version 1.54 g, JAVA 1.8.0_245, 64 bit, NIH, USA) was used in the determination of the droplet size, count, and their distribution. The mentioned software has a specific tool which enables the identification of all droplets within a representative image and, after proper calibration, to compute their diameter.^23,24^ The image analysis procedure using ImageJ is described in detail in Supporting Information, page 3. The statistical analysis was based on the hypothesis that the droplets observed on the focal plane of interest were sufficiently representative of the emulsion; this feature was confirmed by the repeatability of the experiments. Droplet size distributions are presented using histograms with a bin size of 5 μm. Intensity is computed following eq 1. It must be mentioned that droplet sizes smaller than 1.1 μm are not considered in the size distribution because of the limited resolution of the images (the typical resolution of images was 3.6 pixels/μm).

1 $$I=\frac{n_{i}}{n_{total}}\times 100$$

where *I* is the intensity of droplets (%), *n*_i_ represents the count of droplets with respective diameter range, and *n*_total_ is the total count of droplets in the representative image. Based on the assumption of spherical droplets, for several experiments, we also computed the mass of droplets (in μg) according to eq 2. Then, droplet mass distributions with respect to droplets diameter were expressed as mass intensity; see eq 3.

2 $$m=\rho \times \frac{4}{3}\times \pi \times r^{3}$$

3 $$I_{mass}=\frac{m_{i,d}}{m_{total}}\times 100$$

where ρ is the SO density (for SOρ = 0.97 g cm^–3^), *r* is the radius of the respective droplet (cm), *I*_mass_ is the mass intensity of droplets, *m*_i,d_ represents the sum of droplet mass in respective diameter range *d*, and *m*_total_ is the total weight of droplets in the detected sample.

### 2.5 Measurement of Viscoelastic Properties

Shear surface/interface viscoelastic properties describe the resistance of the surface/interface to the shear deformation. The magnitude of this resistance is described using interface complex modulus which is a complex number having two components, interface elastic *G*_i_^I^ and viscous *G*_i_^II^ moduli. The interface complex modulus can be represented either using these moduli *G*_i_^I^ and *G*_i_^II^ or using the absolute value of it |*G*_i_*| and a phase shift δ. Phase shift is a measure of viscoelasticity. In the case of purely elastic interface saving energy, δ = 0°, while in the case of purely viscous dissipative interface, δ = 90°. Brief insight into interfacial viscoelastic properties^17^ is provided in the Supporting Information; see pages 3–5, eqs S1–S8, and Figures S2 and S3.

We measured the interface viscoelastic properties using a rotational rheometer Haake MARS III using a BiCone sensor; see Figure 2, following the methodology recommended by the provider of the rheometer. The details concerning measurement of viscoelastic properties are explained in Supporting Information; see pages 5–8, eqs S9–S16, and Figures S4–S6.

**Figure 2:**
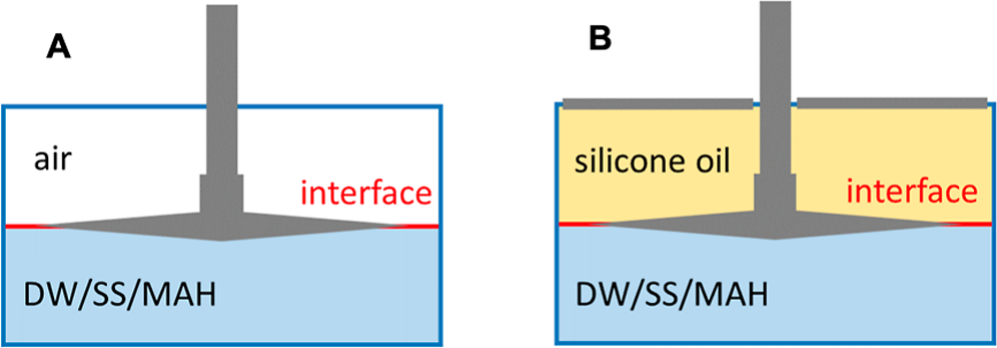
A sketch of the BiCone sensor for measuring interface viscoelastic properties. Viscoelastic properties of the aqueous solution and the air interface (A) and the aqueous solution and oil interface (B).

All measurements were performed at a temperature of 25 °C. Measurements were carried out in oscillatory mode. Prior to each measurement, a pretreatment was performed for 60 s under an amplitude of deformation of 0.001 and a frequency of 0.5 Hz. After the pretreatment, regular measurement was performed with a constant oscillation frequency of 0.5 Hz and deformation amplitude varied 0.001–1 (amplitude sweep).

## 3 Results and Discussion

Since 1990, a wide range of literature has been proposed based on clinical observations of SO emulsification.^25,26^ These qualitative works described complications related to SO emulsions, their management, and possible prevention. Several recent studies have been suggested that the emulsification can be limited by the utilization of high-molecular-weight SO containing long-chain silicone molecules as endotamponade in vitreoretinal surgery.^27,28^ The development of novel long-term hydrogel-based vitreous humor substitutes has also become a recent trend in experimental works.^29–32^ However, until these strategies can be adopted in practice, the only current clinic prevention of the SO emulsification within the tamponing of retinal detachment is the application of highly purified medical-grade SO with a higher viscosity.^16^ Nevertheless, the limitation of the emulsification of high-viscosity SO has not been directly experimentally proven. On the other hand, less viscous SO can be easily injected and removed through smaller cannulas in surgical practice.^16^ In the current literature, only a few papers are devoted to the experimental (in vitro) emulsification tests of SO employed for retinal detachment treatment. SO emulsification with respect to the retinal detachment treatment has been investigated in closed glass vials/cuvettes,^19,27,28,33–36^ via artificial model eyes^6,37–39^ and furthermore, using evolved eye-on-a-chip devices,^40–44^ which are very practical in this way.^3^ Only the above-mentioned research can provide quantitative information on the nature, number, size, and distribution of the emulsified droplets and, therefore, clarify the specific conditions of emulsion formation. Therefore, detailed descriptions of SO emulsification conditions and understanding of this phenomenon are perforce required. Here, we investigate SO emulsification in a glass vial with the aim of readily mimicking hydrodynamic conditions in the eye. This paper is devoted to the influence and interplay of several parameters, such as oil/water ratio, temperature, and composition of the aqueous phase, on SO emulsification.

### 3.1 Emulsification Experiments in the Absence of Proteins

Beginning experiments with DW and SS were performed with the aim of distinguishing the behavior of proteins and excluding the influences of inorganic components present in the human aqueous humor. All mixtures of aqueous solution–SO shaken by a rotary motion in a partially filled vial were emulsified independently of the volume ratio between phases or composition of the aqueous phase.

#### 3.1.1 Coexistence of W/O and O/W Emulsions

During experiments with DW and SS, the separation of the oil and water phase (based on their densities) with simultaneous production of both W/O and O/W emulsions in one vial (Figure 3A) was observed. The production of W/O and O/W emulsions in one vial was proved using the dyeing method with MO. The example of a dyed water droplet in the continuous oil phase is presented in Figure 3B, while the undyed oil droplet in the continuous dyed water phase is depicted in Figure 3C.

**Figure 3:**
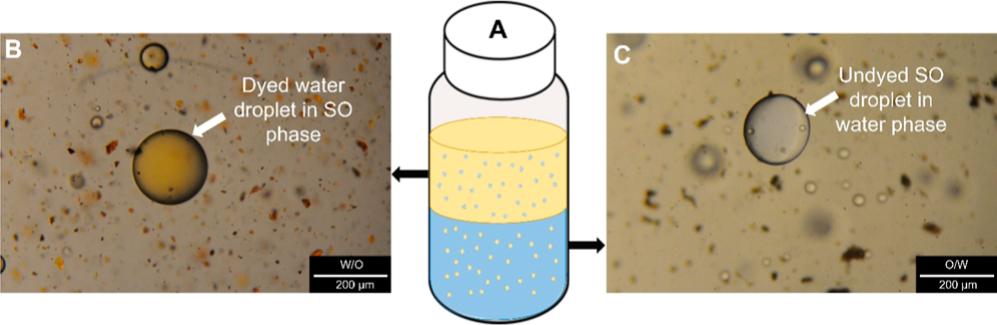
W/O and O/W emulsions produced in one vial during the emulsification experiments (A), water droplet dyed with MO in the oil continuous phase (B), and undyed oil droplet in the dyed water continuous phase (C) (the orange-colored background structures in (B) represent undissolved MO particles dispersed in the continuous oil phase. In the dyeing method, MO dissolves preferentially in the aqueous droplets of W/O emulsions while remaining undissolved in the oil phase).

The separation of two continuous phases with simultaneous production of both W/O and O/W emulsions was observed in all tested conditions. It is in contradiction with some recent emulsification observations which revealed that no emulsions are generated in the absence of proteins.^6,19^ Despite, the two phases with SO on top and aqueous below after mechanical emulsification have been detected in the available literature.^13,27,28,34,35^ Furthermore, several authors^27,28,34^ described that the centrifugation, sonication, or vortex mixing of SO with (distilled) water in the range of 10–120 min can produce O/W or W/O emulsions.

For example, Caramoy et al.^27^ explored the droplets in the bottom of the oil phase, suggesting that it might be W/O. Chan et al.^34^ believed that observation of two phases (oil and water) is mainly caused by the high amount of SO applied (oil/water ratio 1:1). As authors^34^ verbatim wrote: “···not all the oil was dispersed···”. They explained that these results therefore reflect how much SO can be successfully dispersed by mechanical emulsification rather than measuring the resistance of the SO to be emulsified.^34^

Authors^34^ also delineated that the change of the oil/water ratio with a lower amount of SO can produce more consistent results during homogenization, which is an established scientific method for generating emulsions.^45^ In the work of Chan et al.,^34^ all SO in each modification of emulsification test was exhaustively emulsified, leaving no bulk oil. Again, it is in contradiction with our outcomes because we verified the separation of SO and DW (or SS) phases with simultaneous O/W and W/O emulsion formation in each oil/water ratio tested.

The distinctions between our results and those of others can be explained by mechanical homogenization of the oil/water mixtures under different conditions. We put into the system much more energy (shaking within 24 h.) than in other cases.^27,28,34^ We also always collected and analyzed both continuous phases, even though emulsification was not visible (e.g., opalescence of the SO phase or visible oil droplets in the aqueous phase). It is questionable how and whether other scientists collected and analyzed the resulting two continuous phases.

#### 3.1.2 Effect of Salt Presence, Oil/Water Ratio, and Temperature

To comprehensively evaluate the factors influencing SO emulsification, we systematically examined the effects of aqueous phase composition (DW and SS), temperature (20 and 40 °C; the selection of these two specific temperatures is explained in Section 3.2.2.), and oil/water ratios on emulsion formation. These parameters represent key physicochemical and environmental variables that may affect droplet generation, interfacial behavior, and the overall emulsification dynamics under clinically relevant conditions. In all experiments, the average droplet diameter, total count, and size distribution were determined in protein-free systems. The results of droplet size are summarized in Table 2, while the total droplet counts and detailed size distributions are shown in Figures S7–S9. Representative microscopic images are presented in Figure 4A–D.

**Table 2:** Range of Droplet Diameters Observed in W/O and O/W Emulsions across All Oil/Water Ratios under Various Conditions

emulsion type	aqueous phase	temperature/°C	droplet diameter range/μm
W/O	DW	20	1.1–50.8
		40	1.1–61.8
	SS	20	1.7–35.7
		40	1.4–39.2
O/W	DW	20	1.2–76.9
		40	1.2–74.4
	SS	20	1.4–61.8
		40	1.4–61.8

**Figure 4:**
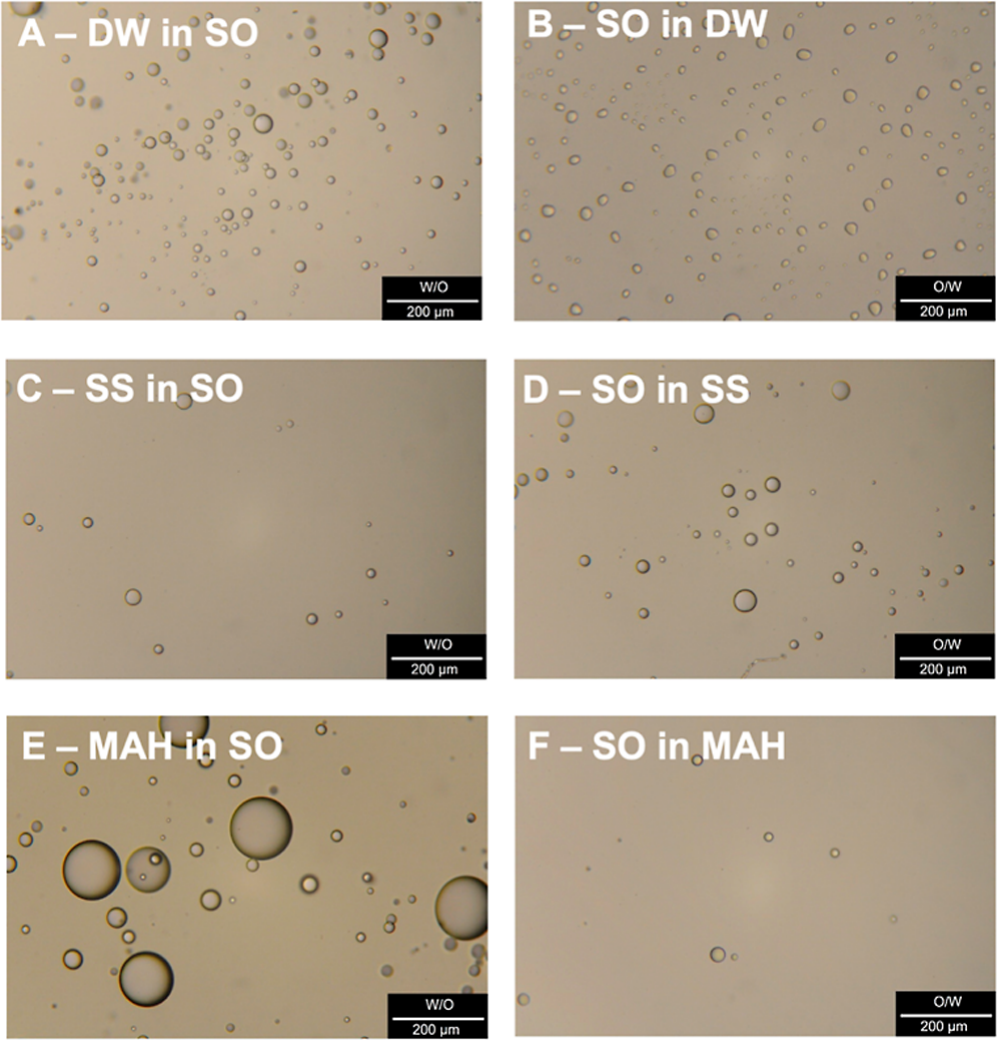
Examples of microscope images of the W/O and O/W emulsions formed within experiments with DW (A,B), SS (C,D), and MAH (E,F) (experimental conditions: oil/water ratio = 5:5; temperature 20 °C).

Overall, droplets exhibited diameters ranging from 1.1 to 70 μm, with the majority (70–80%) between 5 and 20 μm, regardless of temperature, aqueous phase composition, or oil/water ratio. The presence of inorganic salts (SS) produced no substantial differences in total droplet count or size distribution compared with DW, confirming that electrolytes alone have only a minor impact on the aqueous–SO interface. Hence, the influence of the aqueous composition is limited when no surface-active proteins are present.

However, closer comparison of the distributions in Figures S8 and S9 reveals several subtile but important distinctions. Emulsions formed with SS displayed slightly narrower droplet size distributions and a higher proportion of small droplets (<10 μm) compared with DW. This can be possibly attributed to the presence of Na^+^ and Cl^–^ ions, which may slightly reduce interfacial tension and hinder droplet coalescence through electrostatic screening, resulting in marginally finer emulsions.^46^ In contrast, DW systems showed broader size spectra and a higher frequency of coalescence events, producing fewer but larger droplets. The effect of the oil/water ratio was consistent across the two aqueous systems. Increasing the proportion of the aqueous phase progressively shifted the system from W/O to O/W emulsions. Furthermore, the distributions suggest that increasing the ratio of the aqueous phase (DW or SS) compared to that of SO leads to an enhanced intensity of W/O droplets and vice versa. Only the oil/water ratio of 5:5 shows the deviation from the described trend. In the case of DW, the number of W/O and O/W droplets was markedly higher than that when SS was used.

Temperature influenced the emulsification behavior in a phase-ratio-dependent manner. The number of droplets (both W/O and O/W) decreases with increasing temperature (40 °C) for the case of balanced SO/water ratio 5:5, but increases for the case of 3:7 ratio in both DW and SS systems. Although high-viscosity SO exhibits minimal intrinsic changes in viscosity and interfacial tension between 20 and 40 °C,^47,48^ even small thermal variations may significantly affect the dynamic balance between droplet breakup and coalescence.^49,50^ At the 5:5 ratio, the large interfacial area and frequent droplet collisions promote coalescence^51^ when viscosity decreases with temperature, leading to fewer but larger droplets. In contrast, at the 3:7 ratio, the continuous aqueous phase dominates; here, the reduced interfacial tension at 40 °C facilitates droplet breakup during agitation, while the lower collision frequency limits coalescence. As a result, more numerous and smaller droplets are produced. Thus, the opposite temperature trends at 5:5 and 3:7 arise from a shift in the balance between the breakup and coalescence.

In summary, (i) DW and SS systems exhibit almost similar macroscopic behavior, but SS yields slightly smaller droplets due to ionic effects; (ii) increasing the aqueous fraction promotes a transition from W/O to O/W emulsions with smaller droplet sizes; and (iii) temperature modifies emulsification differently depending on the phase ratio through competing effects of viscosity reduction, interfacial tension decrease, and collision dynamics.

#### 3.1.3 Viscoelastic Properties of the Water–Oil Interface in the Absence of Proteins

With the aim to elucidate the complexity of SO emulsification, the interfacial shear viscoelastic properties were investigated in the case of aqueous phases without proteins (DW, SS) in contact with either air or SO. Figure 5 summarizes the measured complex modulus (|*G*_i_*|) and phase angle shift (δ) as functions of shear deformation (γ). These parameters provide insights into the elastic (saving energy) or viscous (dissipative) character of the interface, which is crucial for understanding emulsion formation and complements the results and phenomena described above.

**Figure 5:**
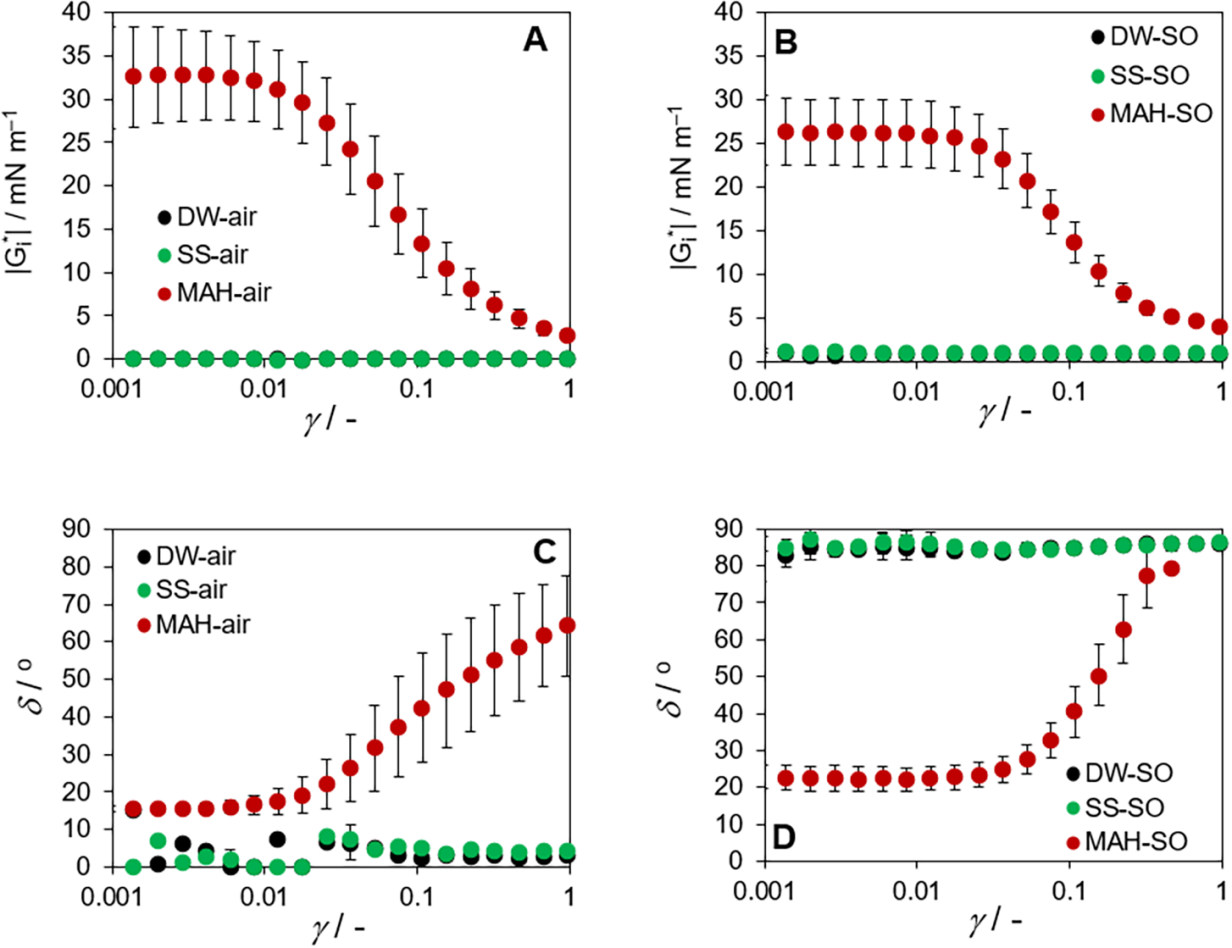
Amplitude sweep variation of viscoelastic properties (dependence of the complex modulus |*G*_i_*| (A,B) and phase shift δ (C,D) on shear deformation γ measured for the interface of the aqueous phases (SS, DW, and MAH) with air (A,C) or SO (B,D)).

The tests indicated that there are no distinctions in measurements with DW and SS, see black and green points in Figure 5. Both DW and SS exhibit very low values of complex modulus at the interface with air and also with SO, Figure 5A and 5B. Nevertheless, they exhibit low values of phase shift (about 5°), i.e., a very elastic interface with air (Figure 5C), while at the interface with SO, they exhibit phase shift about 85°, i.e., it behaves as very viscous and dissipative (Figure 5D).

These results show that, without surface-active components, the aqueous–SO interface behaves mainly as viscous and dissipative, losing energy rather than storing it elastically. This explains the poor stability of the emulsions formed with DW or SS.

### 3.2 Emulsification Experiments in the Presence of Proteins

Further emulsification tests were focused on the influences of the protein presence in the aqueous phase. Previous works^6,19^ indicated that the emulsification results obtained using albumin or γ-globulins were not significantly different. Nepita et al.^19^ also published a similar interfacial tension of SO with an aqueous solution of albumin, γ-globulins, and also its mixture (of albumin + γ-globulins). Based on this, it can be predicted that both proteins behave similarly in emulsification processes. However, none of the available research work investigated the composition of aqueous humor and its role in SO emulsification. For this purpose, we collected the published articles^20,21^ on the composition of aqueous humor and, unlike other papers,^6,19^ we prepared the aqueous solution that simulates real aqueous humor containing inorganic salts and human albumin and γ-globulins (MAH); see Table 1. We are convinced that emulsification tests with individual proteins in various concentrations are not required on one hand, and on the other hand, there is a necessity to examine a complex composition of aqueous humor.

#### 3.2.1 Effect of the Protein Presence and Oil/Water Ratio

Expectedly, the emulsification experiments using MAH as the aqueous phase were distinct from tests with DW and SS. The emulsification of SO in a ratio 5:5 with MAH also revealed the formation of both W/O and O/W emulsions, similar to the case presented in Figure 3, see Figure 4E and 4F. Unlike DW or SS, the diameter of MAH droplets detected in SO and SO droplets detected in MAH at 20 °C ranged differently; see Table 3 and compare with Table 2. Figure 6A presents the droplet size distributions of W/O and O/W emulsions produced at 20 °C in a SO/MAH ratio of 5:5. The presence of proteins in MAH promoted the formation of larger W/O droplets while simultaneously reducing both the number and size of O/W droplets, suggesting a competitive redistribution of dispersed phases.

**Table 3:** Droplet Size and Emulsion Type at Different Oil/Water Ratios at 20 °C

SO/MAH ratio	emulsion type	droplet diameter range/μm
5:5	O/W + W/O	O/W: 2.6–28.1 W/O: 2.8–118.3
9:1	W/O only	3.7–231.8
1:9	O/W only	1.2–15

**Figure 6:**
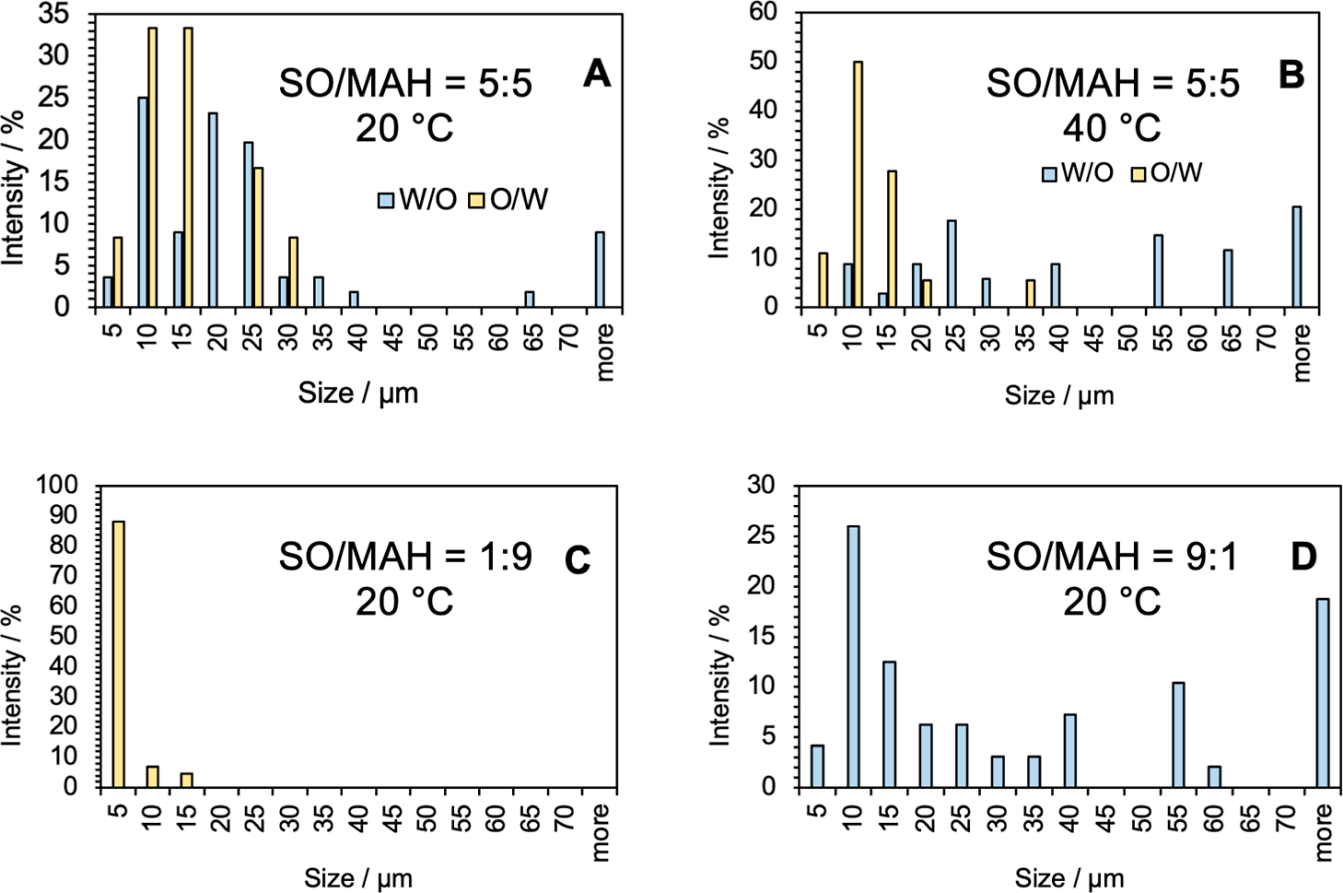
Droplet size distributions (in %) of emulsions obtained through SO emulsification experiments with MAH. Experimental settings: SO/MAH = 5:5, temperature 20 °C (A); SO/MAH = 5:5, temperature 40 °C (B); SO/MAH = 1:9, temperature 20 °C (C); and SO/MAH = 9:1, temperature 20 °C (D).

However, the changes in the SO/MAH ratio to 9:1 and 1:9 furnished completely different outcomes. We observed either W/O or O/W, thereby it was dependent on the SO/MAH ratio. Considering the O/W emulsion generated during the emulsification of the SO/MAH ratio 1:9, droplets of SO <5 μm (including droplets <2 μm) are generally formed (>80% of droplets ≤5 μm); see Table 3 and Figure 6C. The size and distribution of O/W droplets correspond well with those in other works. Whereas Chan et al.^34^ described the most abundant O/W droplets with diameters 1–2 μm, Nepita et al.^6^ detected 6–10 μm with the highest representation. This is probably the crucial finding that mainly influences the emulsification complications connected with retinal detachment; see Section 3.4. Analogously, only the W/O emulsion is produced at a SO/MAH ratio of 9:1. The diameter of MAH droplets observed in SO and its distribution is provided in Figure 6D and the span variation is given in Table 3.

Moreover, unlike the oil/water ratio 5:5, the number of O/W (SO/MAH ratio 1:9) and W/O (SO/MAH ratio 9:1) droplets achieved an elevated number of 43 and 96 in one frame, respectively; see Figure S7 in the Supporting Information. It indicates that not only the presence of proteins in MAH but also the ratio of SO and the aqueous phase strongly influences the emulsion production. In a word, these examinations demonstrate that the lower content of the aqueous phase put into the system caused the emulsification to displace toward the production of W/O only and vice versa.

In addition to the droplet distribution based on the count intensity of the droplets, we also computed the droplet size distribution with respect to the mass of the respective droplets. The total mass of the droplets with diameter >70 μm represents more than 90% of the weight of all MAH droplets detected in SO (oil/water ratio 5:5). However, the experiment with DW performed under the same conditions showed a different mass distribution with almost 50% mass intensity for droplets with a diameter of 20 μm. Detailed results are depicted in Figure S10 in the Supporting Information. The presence of proteins in MAH caused the formation of W/O emulsions with substantially larger droplets, which represented more than 90% of the mass of all droplets detected in this sample. On the other hand, we detected a lower amount of O/W droplets.

In summary, the presence of proteins in MAH significantly influenced both the type, size, and mass distribution of emulsion droplets compared to protein-free systems (DW and SS).

It should be noted that the selected oil/water ratios (1:9, 5:5, and 9:1) were intentionally chosen to represent distinct physiological and pathological conditions during and after the tamponade period. In a normal-sized eye, the vitreous chamber volume is approximately 4 mL; therefore, the 9:1 ratio reflects the postoperative state in which the vitreous cavity is almost completely filled with SO and only trace amounts of aqueous humor remain. The 5:5 ratio simulates transient interfacial regions or localized mixing that may occur due to ocular movements and saccades that intermittently entrain thin aqueous films into the oil layer. Conversely, the 1:9 ratio models conditions of aqueous humor inflow or leakage into the oil phase at later stages of tamponade or during emulsification progression. Collectively, these ratios cover the clinically relevant range of tamponade scenarios—from nearly complete oil filling to advanced aqueous infiltration—where interfacial instability and emulsification are most likely to occur.^6,44,52^

#### 3.2.2 Effect of Temperature

Besides the presence of proteins and the SO/MAH ratio, we considered other features affecting SO emulsification during the tamponade period. In the presence of proteins (MAH), the temperature emerges as one of the possible parameters that slightly affect the size of both W/O and O/W droplets. The size distribution of the droplets in O/W and W/O emulsions produced at 40 °C is presented in Figure 6B.

In SO/MAH at a 5:5 ratio, elevating temperature from 20 to 40 °C shifts the bimodal distribution toward W/O dominance, compare Figure 6A and 6B: the fraction of small O/W droplets (5–15 μm) decreases, while W/O droplets coarsen and populate larger size classes (30–70 μm). This trend likely reflects thermally enhanced protein adsorption and partial denaturation at the MAH–SO interface, which reinforces the viscoelastic interfacial film, which suppresses interfacial deformation and droplet breakup, leading to coarser and more persistent W/O droplets. At the same time, increasing temperature promotes partial protein unfolding and stronger intermolecular interactions at the MAH–SO interface, resulting in a more elastic and cohesive interfacial film. This structural reinforcement reduces interfacial mobility, limiting the rearrangement of adsorbed proteins necessary for new interface formation and thereby suppressing the nucleation of small O/W droplets.

By contrast, in the absence of proteins (DW or SS), the temperature had no significant impact on emulsification. This indicates that temperature-related effects emerge primarily when surface-active proteins are present at the interface, pointing to a protein-mediated modulation of interfacial behavior rather than a purely thermal mechanism.

It should be noted that the experiments were conducted at 20 and 40 °C to cover a wide range of relevant conditions. While typical intraocular temperatures range from 30.1 to 34.7 °C,^53^ the lower limit (20 °C) provided a stable, reproducible laboratory baseline, while the upper limit (40 °C) was chosen to simulate febrile or extreme inflammatory conditions. Although intraocular temperatures may not normally reach 40 °C, body temperatures of ≥ 42 °C have been reported during hyperpyrexia, and ocular temperatures are expected to increase accordingly. Ethical and technical limitations prevent direct intraocular measurements during fever, so this upper boundary is supported primarily by thermal modeling.^54–56^

Choosing 20 and 40 °C instead of narrower physiological intervals (e.g., 32–37 °C) enhanced the contrast and helped identify temperature-dependent trends more clearly. This boundary-testing approach is common in bioengineering to evaluate system sensitivity and allows interpolation across intermediate values, including physiological temperatures. Importantly, high-viscosity SO shows minimal changes in viscosity and interfacial tension between 20 and 40 °C.^47,48^ Thus, our results at these two temperatures likely encompass the full range of emulsification behavior relevant to clinical scenarios.

#### 3.2.3 Viscoelastic Properties of the Water–Oil Interface in the Presence of Proteins

Contrary to the salt presence, which did not influence viscoelastic properties of the water–air and water–SO interfaces, the presence of proteins (MAH) significantly altered these viscoelastic properties. In the case of measurement with MAH, i.e., the presence of proteins, the reproducibility was not very satisfying, see large error bars of red points in Figure 5, but the basic trends are clear. The proteins act as surfactants, strengthening the interface between MAH and both air and SO in terms of complex modulus; see Figure 5A and 5B. In the case of the MAH and air interface, the elasticity does not change largely (compare values of 5° and 15° in Figure 5C), but it is seen that above deformation of 0.01, the interface becomes weaker and more viscous. The most important observation was made in the case of the MAH and SO interface. The presence of proteins (MAH) not only strengthens the interface in terms of the complex modulus, but the phase shift indicates much more elastic behavior in comparison with the DW or SS interface with SO (compare values of 22° and 90° in Figure 5D). In summary, a viscous dissipative interface (DW or SS with SO) changes to strongly elastic and saving energy if proteins accumulate at the interface.

Proteins adsorbed at the MAH–SO interface form a viscoelastic film that increases interfacial elasticity^57^ and resists droplet breakup.^58,59^ This leads to the formation of larger droplets during emulsification, particularly in the W/O emulsion. This behavior likely arises from the protein-induced strengthening of the interfacial film, which increases the elastic modulus, thus possibly favoring the persistence of large W/O droplets within the oil phase. This outcome is consistent with the stabilizing role of interfacial elasticity: while in the oil-continuous phase, it preserves larger aqueous droplets (W/O); in the aqueous-continuous phase, it suppresses excessive oil droplet breakup, resulting in smaller and less numerous O/W droplets. In contrast, in DW and SS, which lack surface-active components, the interface is viscous, see Section 3.1.3 above, resulting in more extensive fragmentation and a higher number of smaller droplets.^60^ Overall, proteins or other interfacial active species are essential to providing elasticity and stability to the SO-aqueous interface.

#### 3.2.4 Effect of Physical Exertion

Although shaking speed directly governs the hydrodynamic shear rate and turbulence intensity in vitro, such controlled conditions cannot replicate the complex, low-frequency, and irregular micromovements of the human eye.^6,14^ Computational and fluid-dynamic models show that saccadic motions generate transient pulsatile shear fields rather than continuous turbulence,^61,62^ confirming that high-speed agitation (≥300 rpm) lacks physiological relevance. At low speeds (≤100 rpm), minimal interfacial disruption can be produced, indicating that such mild agitation cannot overcome the interfacial tension between SO and the aqueous phase. Therefore, 200 rpm was selected as a practical compromise, ensuring reproducible emulsion formation while approximating the energy input of ocular micromovements.

We clearly demonstrated that spontaneous emulsification does not occur in the absence of mechanical agitation, confirming that the formation of stable SO emulsions requires the input of an external mechanical energy. In our experiments, samples maintained under static conditions for several weeks showed no evidence of droplet generation, indicating that interfacial destabilization alone is insufficient to induce emulsification. This finding strongly supports the view that intraocular emulsification in vivo is primarily driven by hydrodynamic forces associated with eye movements rather than spontaneous interfacial instability.

Physiologically, saccadic and microsaccadic eye motions induce localized shear and recirculation zones within the vitreous cavity, as confirmed by computational fluid dynamics models.^61,62^ These intermittent mechanical stresses may accumulate over time, especially during repetitive or vigorous eye movements, facilitating gradual droplet formation once the interfacial protein film becomes sufficiently deformed. Consequently, periods of intense or prolonged physical activity followed by rest could transiently enhance intraocular shear gradients and promote the emulsification onset. Although this interpretation is based on indirect experimental evidence, it aligns with both rheological theory and clinical observations reporting higher emulsification incidence in active or younger patients.^63^

Therefore, the present results reinforce the concept that SO emulsification is an energy-driven, shear-dependent process rather than a spontaneous event and that its likelihood is modulated by the dynamic mechanical environment of the eye.

#### 3.2.5 Emulsion Stability

Afterward, we kept the formed emulsions under static conditions at 20 °C for 4 months. The results of the stability of emulsions are given in Table S1 in the Supporting Information. In contrast with DW and SS, which provided small-scale changes in droplet count and size after 2 months, the emulsions formed with MAH did not report any considerable changes in droplet count and size after 4 months, which shows that the emulsions are stable for a long time period.

The stability of formed emulsions within 90 days also confirms the work published by the research group of Nepita et al.^6,19^ It should be mentioned that this situation is undesirable because stabilizing emulsions over a period of months prolongs the duration of health complications associated with this state.^7^ The observed stability of MAH-based emulsions over several months suggests that once emulsified, droplets can persist for extended periods under static conditions. Although the ocular environment is more dynamic, clinical reports have shown that residual SO microdroplets may remain in the vitreous cavity or anterior chamber long after tamponade removal, potentially contributing to sustained inflammatory or pressure-related complications.^8–10^ Therefore, our in vitro results may reflect a risk of the long-term persistence of emulsified particles in vivo, underscoring the need for further studies on their clearance dynamics.

### 3.3 Effect of Interfacial Viscoelasticity on Emulsion Behavior

The viscoelastic behavior of the aqueous–SO interface has clear implications for the type and stability of emulsions formed under mechanical stress. Recently, it has been confirmed that the presence of proteins in MAH in contact with SO induces a significant decrease in interface tension.^6,19^ On the other hand, the presence of proteins strengthens the water–SO interface, and it promotes an elastic behavior. This interfacial elasticity is known to influence droplet formation: elastic interfaces tend to resist deformation and recover their original shape after stress, while viscous interfaces dissipate energy and break more readily into smaller droplets.

In the case of the O/W emulsions, the dispersed SO droplets originate when portions of the oil phase detach under mechanical stress. The presence of proteins in MAH facilitates this process by rapidly adsorbing at the emerging interface and lowering the interfacial tension, thereby allowing the oil phase to fragment into smaller droplets under mild shear. Once adsorbed, the same proteins stabilize these droplets against coalescence by forming a thin viscoelastic film, which limits further breakup. Consequently, small but relatively stable O/W droplets are observed, as exemplified by the SO/MAH ratio of 1:9 (see Figure 6C).

Conversely, in W/O systems where MAH droplets are dispersed within a continuous SO phase, the adsorbed proteins form a more cohesive and elastic interfacial film that resists deformation. This elastic strengthening suppresses droplet breakup during agitation and promotes the persistence of large aqueous inclusions within the oil.^34^ As a result, fewer but larger W/O droplets are produced, consistent with our observations at SO/MAH ratios of 5:5 and 9:1 (Figure 6A and 6D).

Thus, the simultaneous formation of small O/W and large W/O droplets arises from the dual role of interfacial proteins: their ability to lower interfacial tension during droplet generation facilitates O/W formation, while their capacity to enhance interfacial elasticity after adsorption stabilizes large W/O droplets against further breakup.

Taken together, the interfacial viscoelastic measurements correlate well with the observed emulsion morphology and provide a mechanistic explanation for the dual formation of both emulsion types. At balanced SO/MAH ratios (e.g., 5:5), sufficient quantities of both aqueous and oil phases allow for mutual dispersion during mechanical agitation, resulting in simultaneous formation of W/O and O/W emulsions. Each phase can act as either continuous or dispersed depending on local shear forces and interfacial conditions.^58^

In a word, this dual formation of small O/W and large W/O droplets under balanced SO/MAH ratios (5:5) results in a bimodal size distribution. It probably arises from the coexistence of two interfacial mechanisms: elastic protein films in MAH stabilize large aqueous droplets (W/O), while localized phase inversion and incomplete interfacial coverage allow the formation of smaller oil droplets in the aqueous phase (O/W); also see Section 3.2.

However, at asymmetric ratios, such as 1:9 or 9:1, the dominant phase typically forms the continuous medium, restricting the emulsification to one emulsion type. Specifically, at 9:1 (SO/MAH), the continuous SO phase favors W/O emulsions with dispersed aqueous droplets, while at 1:9, the continuous aqueous phase favors O/W emulsions with dispersed oil droplets. This aligns with classical emulsion theory, where volume ratio and interfacial properties dictate emulsion type, making simultaneous formation of both types energetically unfavorable or physically unfeasible under these conditions.^58^

In contrast, protein-free systems (e.g., with distilled water or saline solution) exhibit a weak, unstable interface lacking surface-active agents.^60^ Under mechanical shear, both phases fragment randomly without selective stabilization, allowing both W/O and O/W emulsions to form simultaneously, even at highly asymmetric phase ratios. The absence of interfacial elasticity or surface tension gradients means that there is no barrier to reciprocal droplet formation.

Thus, the presence of proteins stabilizes the interface,^57^ promoting selective formation of a single emulsion type depending on the dominant continuous phase, whereas protein-free systems allow coexistence of both emulsion types due to interfacial instability.

### 3.4 Possible Fate of W/O and O/W Droplets

In theory, the environment in the vitreous chamber is not entirely consistent due to eye movements,^64^ and therefore the specific variable conditions, which have not yet been sufficiently described, can theoretically lead to the simultaneous production of both O/W and W/O emulsions. In fact, it depends on the presence of proteins in MAH, oil/water ratio, temperature, and physical movements. We suggest the state in which production of both types of emulsions occurs as follows. The flow of aqueous humor^65^ in a thin aqueous film causes the entrainment of small SO droplets into aqueous film and the O/W emulsion is formed. Simultaneously, the aqueous mass passes through the elastic interface into SO, and large W/O droplets with a higher diameter are produced.

Although the description of W/O emulsions in the current literature is rare, the production of W/O droplets is supported by the analysis of SO removed from the vitreous cavity after a short-time tamponade of retinal detachment. Microscopic analysis of real samples of medical-grade SO removed from the vitreous cavity revealed the presence of W/O emulsions formed during the tamponade of a retinal break.

Figure 7A displays a microscope image of the SO sample with water droplets removed from the vitreous cavity. The total count of water droplets in the SO phase reached 8 in a representative image, while their diameter ranged from 10.7 to 79.8 μm. The other 2 samples of SO removed from vitreous cavities provided comparable results in dimensions and quantity of droplets. It should be noted that the MO dying method was used to verify the identity of aqueous droplets dispersed in the SO phase. MO selectively colors the aqueous phase, producing orange coloration of water droplets, whereas undissolved MO particles remain visible in the SO. Representative MO-dyed images from in vitro experiments for all 3 patients are provided in Figure S12 in the Supporting Information to illustrate this verification principle.

**Figure 7:**
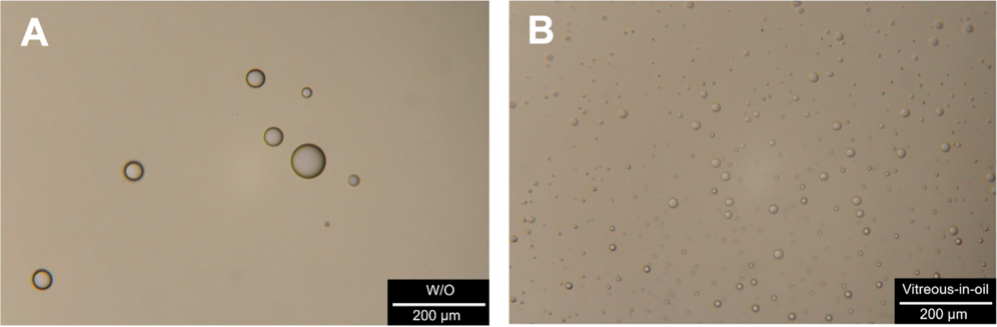
Examples of microscope images of the water droplets dispersed in medical-grade SO recovered from vitreous cavity (A) and of the porcine vitreous humor droplets dispersed in commercial-grade SO (B) (the microscope images verified the W/O emulsion by the dying method with MO are provided in Figure S12 in Supporting Information).

It must be mentioned that the intensity and size of droplets cannot be completely compared to those of the W/O droplets produced in glass vials (compare Figures 4E and 7A). Notwithstanding, the trend of larger droplets still remains. It must be noted that the clinical samples (*n* = 3) included in this study were provided as an illustrative proof of concept to demonstrate that W/O emulsions can also occur in vivo. These cases were not intended to support epidemiological conclusions. A comprehensive clinical study is currently being designed to investigate the correlation between the degree of emulsification and postoperative outcomes such as intraocular pressure changes, anterior chamber migration, and inflammatory signs. Additionally, further clinical cases involving the detection of water droplets in explanted SO have been reported by our collaborators. These cases are now being systematically collected and will form the basis of a dedicated study focused on evaluating the incidence and clinical implications of this phenomenon.

All of these findings prompt further inquiry into the potential clinical implications of W/O emulsions, raising two fundamental questions: (i) What and if are there any health complications associated with the formation of W/O emulsions? (ii) How do these emulsions affect the simultaneous formation of the O/W droplets and their further fate? The health complications associated with the production of W/O emulsions have not yet been described in the literature, and further examinations are thus required. However, it is clear that the production of W/O droplets is a completely undesirable phenomenon and should be avoided. Possibly, W/O droplets may interfere with the visual function by disrupting optical clarity.

On the other hand, the formation of small O/W droplets is supported by recently described break ups of the SO into droplets and its dispersion in a thin layer of water near the retina. SO droplets in the mentioned thin aqueous film may migrate from the vitreous cavity to the anterior chamber;^12,13^ see Figure 8. In 1996, this hypothesis was confirmed by Savion et al.^66^ They reported the presence of emulsified SO droplets in the anterior chamber.^66^

**Figure 8:**
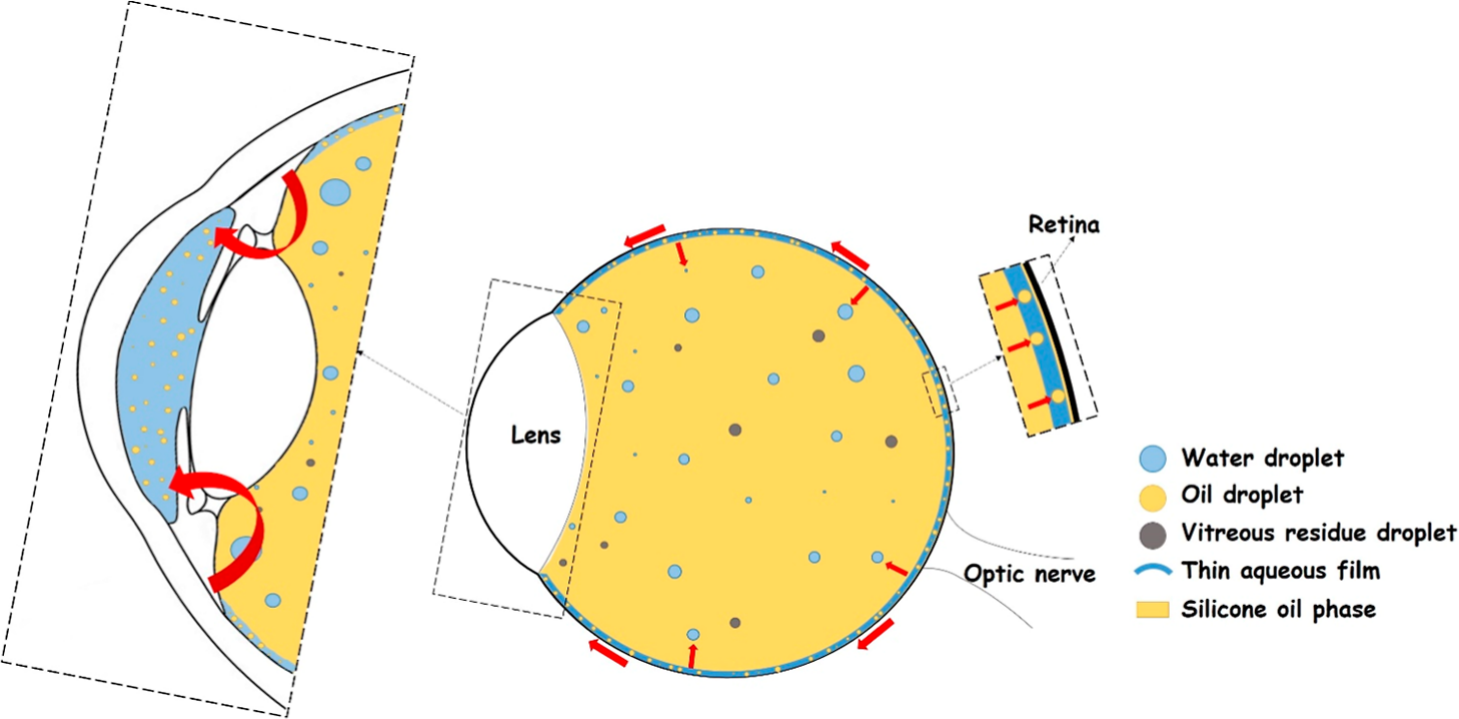
Simultaneous phenomenon of SO emulsification within retinal detachment complications (for a detailed structure of the eye; see Figure S11 in the Supporting Information).

Several clinical and histopathological studies have reported that emulsified SO droplets can trigger ocular inflammation through macrophage activation and cytokine release in the surrounding tissues.^7–10^ In this study, we focused primarily on the physicochemical mechanisms of emulsification rather than biological outcomes. Therefore, the possible link between droplet formation and inflammation is discussed here only as a literature-based hypothesis. Future studies combining in vitro ocular cell models and interfacial chemistry will be required to establish the immunological relevance of emulsified particles.

However, based on other studies, the distribution and sizes of O/W droplets led us to the following theory. The O/W droplets in the anterior chamber may be able to migrate through a trabecular meshwork that is made up of three layers with different pores^67^ which can play a role in the separation of droplets based on their size. Through the third layer of the trabecular meshwork, Juxtacanalicular tissue, only O/W droplets in the range 0.5–2 μm could theoretically pass.^67,68^ The next path of the drops would then be through the Schlemm channel to the sclera;^69^ see Figure 9. Because the Schlemm canal collects aqueous humor from the anterior chamber and delivers it into the episcleral blood vessels via aqueous veins, the presence of SO droplets is highly undesirable in this area and may further cause health complications. However, this interpretation remains a theoretical consideration based on droplet size and literature data, and no biological validation was performed in this study. This assumption represents a working hypothesis that will be experimentally verified using our artificial trabecular meshwork model (see Section 3.5).

**Figure 9:**
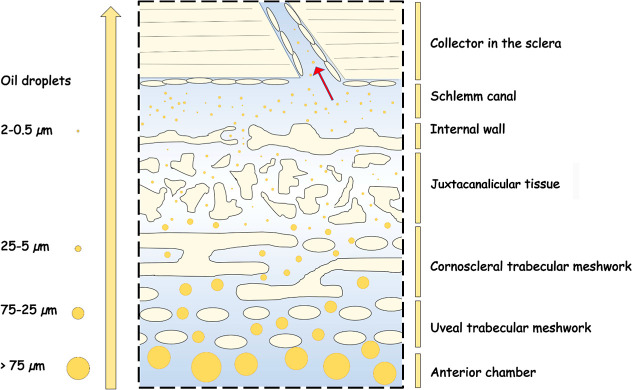
Migration of SO droplets through a trabecular meshwork.

Finally, an emulsified vitreous humor should also be considered. Earlier, several authors^70,71^ reported that during vitrectomy, vitreous humor residue can remain in the vitreous cavity. Interestingly, only Nakamura et al.^13^ in 1990 mentioned the production of liquefied vitreous humor droplets in SO. Since then, the production of the mentioned emulsion has not been further investigated, and the possible complications with the mentioned phenomenon have not been described.

Therefore, the emulsification experiment with porcine vitreous humor was done with the aim of simulating the behavior of liquid vitreous humor residue. When SO was shaken in a vial with a liquified porcine vitreous humor, an emulsion was formed. This emulsification was associated with dispersion of porcine vitreous humor in the continuous phase of SO; see Figure 7B. The SO/vitreous ratio in this experiment was approximately 500:1, corresponding to a strongly oil-dominated system that mimics the residual vitreous content after vitrectomy.^70,71^ Because a single homogeneous emulsion phase was produced under these conditions, it can be unambiguously concluded that the continuous phase was SO with dispersed vitreous droplets. Furthermore, the vitreous-in-oil emulsion was verified by the dying method with MO (data not shown).

The total count of vitreous droplets in the SO phase reached 181 in a representative image, while their diameter ranged from 1.1 to 20.8 μm. The detailed distribution of vitreous droplets in the oil continuous phase is presented in Figure S13 in the Supporting Information. In summary, we observed the intensive production of vitreous humor droplets in SO with a typical size of 20 μm. This ability of production of this emulsion is in good agreement with above-mentioned ref 13. However, the influence of vitreous humor droplets in SO and its side impacts are questionable. Therefore, further chemical engineering and clinical studies related to the impact of vitreous droplets are required.

### 3.5 Limitations and Future Research

Despite all of our findings, there are several limitations in the present study. First, our research group also plans to perform the emulsification experiments with a SO5000 with a viscosity of 4990 mPa·s. Interestingly, our preliminary measurements of viscoelastic properties of this SO5000 provide the opposite trend that the literature described.^16^ The interface between MAH and SO with a viscosity of 4990 mPa·s showed a less elastic, i.e., more dissipative behavior, in spite of this interface being a little more rigid in terms of complex modulus than that of SO1000 with a viscosity of 971 mPa·s; see Figure 10. In competition between elastic structure of proteins and viscous/dissipative fluids, the more dissipative behavior of the MAH–SO5000 interface can be attributed to the higher contribution of the internal friction of SO5000 to interface due to five times higher viscosity than that of SO1000. It possibly indicates that the higher viscosity has no strict impact to prevent the SO emulsification. This also contributes to our effort and the necessity to accurately describe the emulsification of SO in vitro. Therefore, more extensive tests are required to investigate SO emulsification, and this is not our last publication related to this topic.

**Figure 10:**
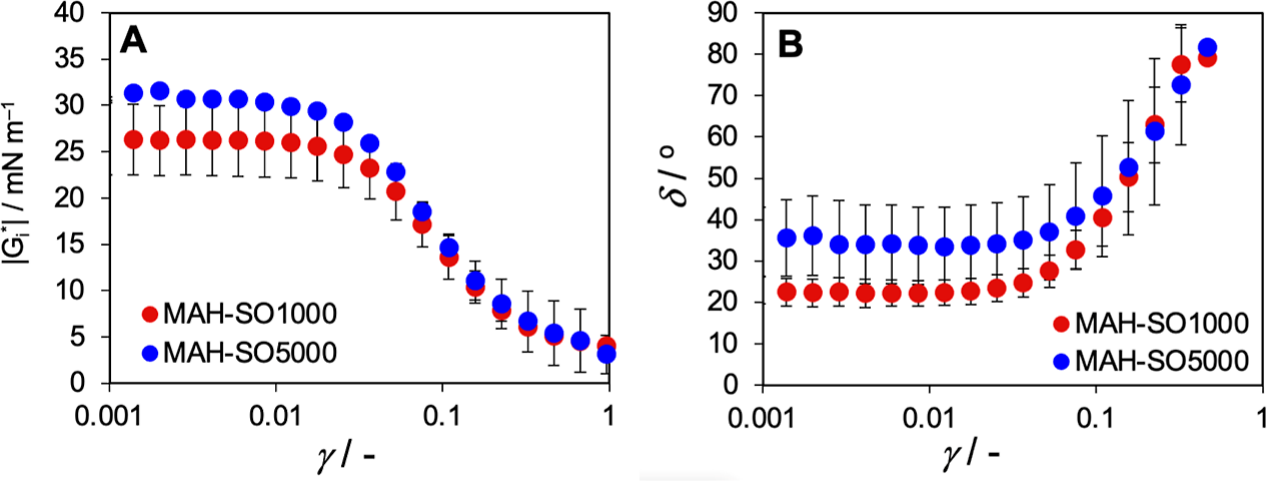
Amplitude sweep variation of viscoelastic properties for SO1000 and SO5000 with a viscosity of 971 and 4990 mPa·s. Dependence of the complex modulus |G_i_*| (A) and phase shift δ (B) on shear deformation γ measured for the interface of MAH with silicone oil.

Second, we want to clarify in detail the production of both O/W and W/O emulsions under conditions more mimicking the reality. Consequently, we intent to design an evolved microscale device, the so-called eye-on-a-chip, for simulating real emulsification condition and/or saccadic movements in the eye. We want to compare the mechanical emulsification via vortex mixing/shaking, microscale-based device, and entrainment of droplets at the MAH–SO interface.

Third, the O/W droplet pathway is only a hypothesis, and we plan to simulate the migration of the O/W droplets through the developed artificial trabecular meshwork. Direct biological modeling of this process would be technically challenging, as maintaining viable ocular tissues with preserved microarchitecture and physiological outflow conditions ex vivo is highly demanding and difficult to reproduce quantitatively. Therefore, we aim to construct an artificial trabecular meshwork model that mimics the structural and hydraulic characteristics of the native tissue, allowing for controlled and repeatable investigation of droplet transport under physiologically relevant pressure gradients.

And last, but not least, the possible impact and complications of W/O emulsions are important points in our future research. The number of clinical samples analyzed here is limited (*n* = 3) and therefore does not allow for estimation of the incidence of W/O emulsions or robust correlation with postoperative complications. The primary aim of including these samples was to confirm that W/O droplets can form in real postoperative oil. Larger, prospective clinical studies are required to quantify frequency and to examine associations with clinical end points.

## 4 Conclusions

The emulsification of silicone oil (SO), widely used as an intraocular tamponade in retinal detachment treatment, is an undesirable phenomenon linked to complications, such as intraocular inflammation, elevated intraocular pressure, glaucoma, and corneal or retinal changes. This study aimed to clarify the in vitro formation of SO emulsions under various conditions, focusing on the interfacial behavior at the aqueous–SO phase interface, particularly the role of protein-mediated biointerfaces. The findings may help to develop strategies to reduce or control intraocular emulsification.

We demonstrated for the first time the simultaneous formation of both O/W and W/O emulsions in vitro. Inorganic salts showed no effect on emulsification behavior or the viscoelastic properties of the water–SO interface. In contrast, the presence of proteins in model aqueous humor (MAH), notably albumin and γ-globulins, significantly influenced emulsion formation, producing small O/W (<2 μm) and large W/O (>70 μm) droplets. Viscoelastic measurements confirmed a more elastic and stronger interface between MAH and SO in comparison with DW, supporting the role of proteins in stabilizing the interface. The occurrence of W/O droplets was further validated by analyzing SO extracted from the vitreous cavity.

The oil/water ratio had a substantial effect: decreasing the oil phase favored the formation of an O/W emulsion, while decreasing the aqueous phase led to W/O emulsions. Importantly, protein content in MAH markedly prolonged emulsion stability, extending it to several months, an undesirable condition due to increased risk of persistent postoperative complications.

While elevated temperature (e.g., from systemic inflammation or illness) had only a limited effect on emulsification, mechanical forces such as turbulence and shear significantly enhanced it. No spontaneous emulsification occurred; movement is necessary to induce droplet formation. Physical exertion followed by rest was found to increase the emulsification risk. Additionally, even when using SO with a higher viscosity (SO5000 instead of SO1000), viscoelastic measurements revealed that the protein-rich MAH–SO interface exhibited less elastic behavior, suggesting that increasing SO viscosity alone does not prevent emulsification. We also found that residual liquefied vitreous humor remaining after vitrectomy can form numerous small droplets in the SO.

Furthermore, we propose that oil droplets formed in the vitreous cavity may migrate to the anterior chamber, then through the trabecular meshwork (potentially acting as a filter), and further via Schlemm’s canal to the sclera and episcleral blood vessels. While this study emphasizes the impact of the O/W droplets, W/O emulsions and emulsions with vitreous humor may also contribute to complications, potentially interfering with optical clarity and visual function.

In conclusion, SO emulsification is a complex process influenced by interfacial protein interactions, oil/water ratios, the temperature, and mechanical forces. The findings challenge existing assumptions, as no prior in vitro studies of SO emulsification have linked such mechanisms with retinal detachment complications. Based on this work, ophthalmologists should note that (i) SO viscosity has a limited effect on emulsification; (ii) patients should avoid prolonged or intense physical exertion; and (iii) elevated temperature caused by hyperpyrexia may be a contributing risk factor. Emulsification-related complications are not isolated phenomena but result from the interplay of multiple conditions affecting postoperative outcomes and treatment success.

## Supplementary Material



## Notes

Diagram of variants of emulsification experiments; experimental procedures for droplet size analysis using ImageJ software; theoretical background and measurement protocol for interfacial viscoelasticity; total droplet counts and size distributions for DW, SS, and MAH at various temperatures and phase ratios; structure of the eye; representative images of SO samples recovered from patients; vitreous humor emulsification experiments; and comparison of emulsion stability (PDF)
