# Supplementary material for: Emulsification Complexity of Silicone Oil in Retinal Surgery: In Vitro Insights into Phase Behavior
Source: ACS Omega. 2026 Jan 9;11(3):4201–15. doi: 10.1021/acsomega.5c09041 (PMC12854598; doi:10.1021/acsomega.5c09041)
Supplement: Supplementary file 1 [file ao5c09041_si_001.pdf]

# SUPPORTING INFORMATION

# Emulsification Complexity of Silicone Oil in Retinal Surgery: In Vitro Insights into Phase Behavior

*Barbora Kamenická[1], Věra Pěnkavová[1*], Eliška Lyko Vachková[1,2], Sandra Orvalho[1], Mária Zedníková[1,2], Natalie Jaklová[1], Petr Stavárek[1], Monika Reháčková[3,4], Patrik Rajs[3,4], Miroslav Veith[3,4], Petr Klusoň[1,5]*

[1]Institute of Chemical Process Fundamentals of the Czech Academy of Sciences, Rozvojová 135, 165 00 Prague, Czech Republic

[2]University of Chemistry and Technology Prague, Technická 5, 166 28 Prague, Czech Republic

[3]Ophthalmology Clinic of the University Hospital Královské Vinohrady, Šrobárova 50, 100 34 Prague, Czech Republic

[4]Third Faculty of Medicine, Charles University, Ruská 87, 100 00 Prague, Czech Republic

[5]Institute for Environmental Studies, Faculty of Science, Charles University in Prague, Benátská 2, 110 00 Prague, Czech Republic

* Corresponding author: Tel: +420 220390222. E-mail: penkavova@icpf.cas.cz

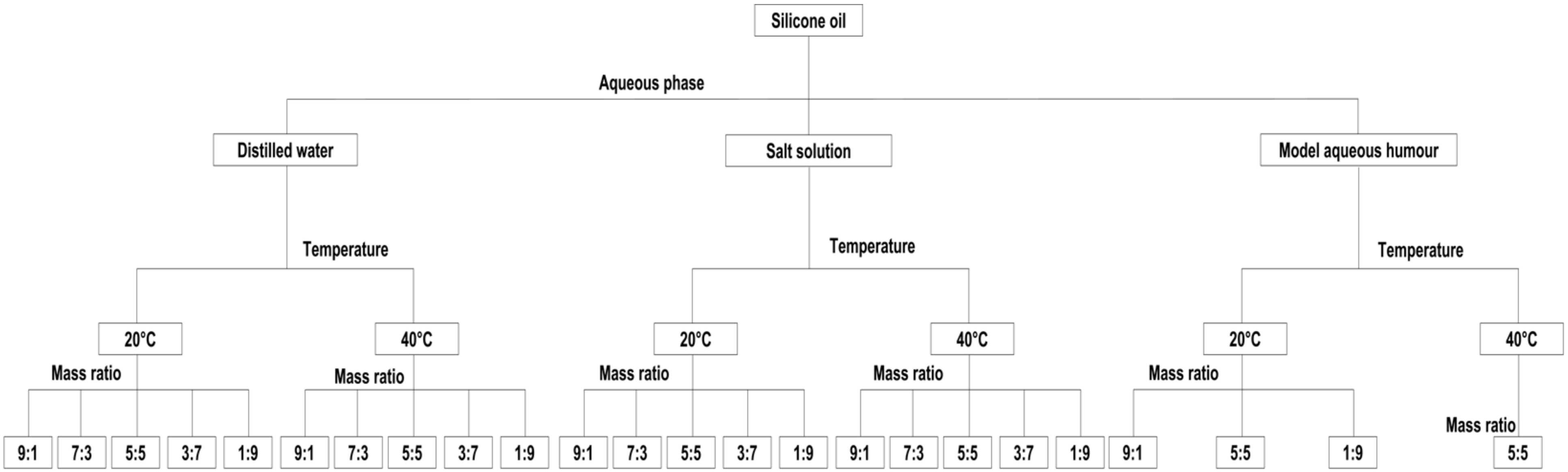


**Figure S1.** Variants of emulsification experiments performed at 200 rpm.

***Image analysis:***

Droplet size and distribution were determined using ImageJ software (version 1.54g, NIH, USA). Color images were analyzed using the "Color Threshold" tool to segment the droplets from the background based on hue, saturation, and brightness parameters. Before analysis, each image was calibrated using the embedded scale bar via the “Set Scale” function to ensure accurate size measurements. Then, we used the “Measure” tool in “Analyze” section, which provided data on droplet count and individual droplet area. Assuming spherical symmetry in 2D, the equivalent diameter was calculated from the area. The resulting data were used to generate droplet size distributions.

***Brief insight into bulk and interface viscoelastic properties*:**

Viscoelastic properties can be characterized in terms of elastic and viscous moduli. In principle, an elastic material can be deformed if shear stress is applied while a liquid material flows under the same conditions, see Fig. S2. The deformation $\gamma$ is described as ratio of elongation $s$ to the material thickness $h$ and the flow is described via shear rate $\dot{\gamma}$, i.e. time change of deformation $d\gamma/dt$ which can be calculated from speed of upper layer $U$ and gap thickness $h$:

$$\gamma = \frac{s}{h} \quad \text{and} \quad \dot{\gamma} = \frac{d\gamma}{dt} = \frac{U}{h} \qquad \text{(S1)}$$

A ratio between applied shear stress and resulting deformation or shear rate is represented *via* Hook law in the case of elastic material (equation S2a) and *via* Newton’s law in the case of liquids (equation S2b):

$$\sigma = G\,\gamma \quad \text{and} \quad \sigma = \eta\dot{\gamma} \qquad \text{(S2)}$$

Here, $G$ and $\eta$ are material properties which describes an internal friction of materials (in other words material resistivity to deformation and flow), i.e. rigidity modulus and dynamic viscosity, respectively.

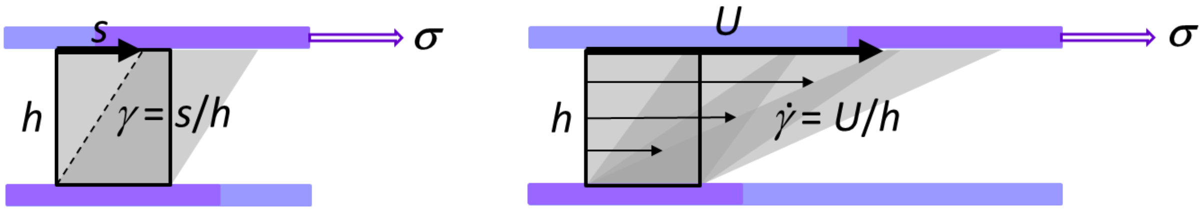


**Figure S2.** A sketch of a deformation (on the left) and flow (on the right) of material in the case of simple shear.

In the case of viscoelastic materials, the behaviour under shear is a mixture of the elastic and viscous properties, and the material property describing the material resistivity to deformation and flow becames a complex number, see Fig. S3. This can be detected under shear in oscillatory regime. If material is subjected to oscillatory deformation of amplitude $\gamma_0$ and frequency $f$ which corresponds to angular speed $\omega = 2\pi f$, than resulting waveform of deformation corresponds to:

$$\gamma(t) = \gamma_0 \sin \omega t \qquad \text{(S3)}$$

and corresponding waveform of shear rate is:

$$\dot{\gamma}(t) = \gamma_0 \omega \cos \omega t \qquad \text{(S4)}$$

Measured response of waveform of shear stress corresponds to nature of tested material, where $\delta$ is a phase shift:

$$\sigma(t) = \sigma_0 \sin(\omega t + \delta) \qquad \text{(S5)}$$

The phase shift $\delta$ ranged from 0° to 90° for a viscoelastic material. In the case of purely elastic material, the shear stress $\sigma(t)$ is in phase with deformation $\gamma(t)$ according to Hook's law and measured phase shift $\delta = 0°$. In the case of purely viscous material, the stear stress $\sigma(t)$ is in phase with shear rate $\dot{\gamma}(t)$, according to Newton's law and measured phase shift to the deformation $\gamma(t)$ corresponds to $\delta = 90°$.

The ratio of amplitude of shear stress $\sigma_0$ and deformation $\gamma_0$ gives information on rigidity of material in the term of absolute value of complex modulus $|G^*|$ and the phase shift $0 < \delta < 90°$ gives information about the proportion of viscoelastic properties. Again, phase shift 0° proves elastic material and associated with storage of energy on one hand, and on the other hand, phase shift 90° indicate viscous (liquid) material and this is connected with energy dissipation. The amplitude ratio of stress and strain gives the complex modulus $G_i^*$ (N m$^{-1}$), which is split into two components through the phase shift: (i) the in-phase component $G_i^I$ (the real part of the complex modulus, referred to as the storage or elastic modulus) and (ii) the out-of-phase component $G_i^{II}$ (the imaginary part of the complex modulus, referred to as the loss or viscous modulus); see Eqs. (S6–8). The $G^I$ is an elastic modulus and $G^{II}$ is an viscous modulus, see Fig. S3.

$$|G^*(\omega)| = \frac{\sigma_0}{\gamma_0} \qquad \text{(S6)}$$

$$G' = \frac{\sigma_0}{\gamma_0}\cos\delta \ \text{ and } G'' = \frac{\sigma_0}{\gamma_0}\sin\delta \qquad \text{(S7)}$$

$$G^*(\omega) = G' + iG'' \ \text{ and } \tan\delta = \frac{G^{II}}{G^{I}} \qquad \text{(S8)}$$

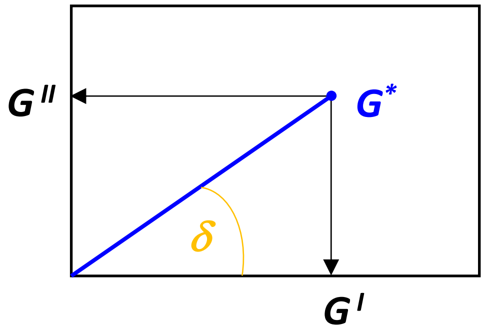


**Figure S3.** An interpretation of viscoelastic properties in the term of complex modulus $G^*$ and phase shift $\delta$ which corresponds to the components of elastic $G^I$ and viscous $G^{II}$ moduli.

Finally, if we measure the interface viscoelastic properties of interphase instead of that of bulk material (the case mentioned above), we change from 3D problem to 2D. Therefore, certain changes are made in units of measured quantities. In 3D problem, the shear stress corresponds to force applied on surface, i.e. units are $N/m^2$ = Pa and the same unit represents the complex $|G^*|$, elastic $G^I$ and viscous $G^{II}$ moduli. In 2D problem, the shear stress corresponds to force applied on line, i.e. units are N/m = Pa.m and the same unit represents the interface complex $|G_i^*|$, elastic $G_i^I$ and viscous $G_i^{II}$ moduli.

***Measurement of interface viscoelastic properties*:**

In the case of rotational interface viscometry, to principial quantities are measured. Applied oscillatory torque $M$ of angular frequency $\omega$, and the resulting angular displacement of the BiCone sensor $\theta$ (see Fig. S4) and the phase shift $\delta$ between $M$ and $\theta$ change are monitored over time $t$:

$$M(t) = M_0\sin(\omega t)\,,\ \theta(t) = \theta_0\sin(\omega t + \delta). \qquad \text{(S9)}$$

$M_0$ and $\theta_0$ corresponds to amplitudes of the torque and the angular displacement.

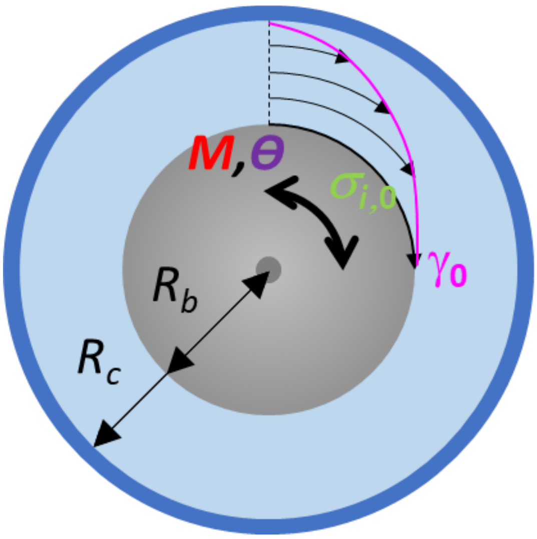


**Figure S4.** A sketch of interface shear deformation in rotational rheometer. $M$ is torque, is $\theta$ angular displacement of the rotor, $\sigma_{i,0}$ is amplitude of interface shear stress, $\gamma_0$ is amplitude of deformation and $R_b$ and $R_c$ are the radius of the BiCone and the cup respectively.

The amplitudes of the interfacial shear stress $\sigma_{i,0}$ in unit Newton per meter and the unitless shear deformation of the interface $\gamma_0$ can be calculated from the monitored quantities ($M_0$ and $\theta_0$) and the geometrical parameters of the sensor ($R_b$ and $R_c$), i.e. the radius of the BiCone and the cup, respectively, just $M_0$ have to be corrected:

$$\sigma_{i,0} = \frac{M_{i,0}}{2\pi R_b^2}, \ \gamma_0 = \theta_0 \frac{2R_c^2}{R_c^2 - R_b^2}. \quad \text{(S10)}$$

Here, $M_{i,0}$ corresponds the interface drag force. In the case of BiCone geometry, the total drag force is given by a sum of the interface and bulk contributions, and $M_0$ must be corrected to give $M_{i,0}$ (the torque corresponding to the bulk contribution should be subtracted). After correction on bulk drug forces, the final recalculation to the complex modulus $G^*$ and its components, elastic $G^I$ and viscous $G^{II}$ moduli can be done:

$$\frac{\sigma_{i,0}}{\gamma_0} = G_i^* = G_i^I + iG_i^{II} \quad \text{(S11)}$$

$$G_i^I = \frac{\sigma_{i,0}}{\gamma_0} \cos\delta, \ G_i^{II} = \frac{\sigma_{i,0}}{\gamma_0} \sin\delta \quad \text{(S12)}$$

The supplier of the Haake MARS III rheometer, Thermo Scientific, recommends in User Guide of BiCone sensor, a method based on calibration measurements for determining of interface viscoelastic

properties. This method is based on comparative measurements with the BiCone sensor fully immersed in test liquid A (lower, heavier phase – here water or aqueous solutions) and in liquid B (upper, lighter phase – here silicone oils), followed by a measurement with the BiCone probe placed at the interface, see Fig. S5 and S6.

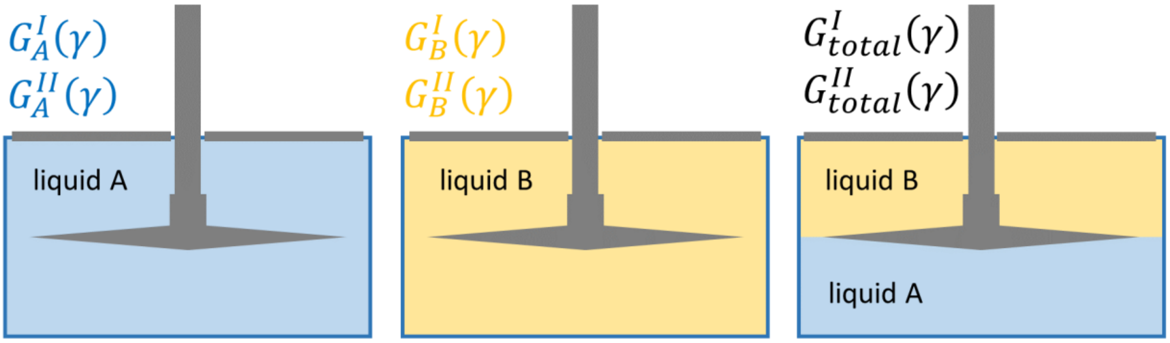


**Figure S5.** Measurement of interface viscoelastic properties - method based on calibration measurements: measurement with liquid A, liquid B and at the interface between two immiscible liquids.

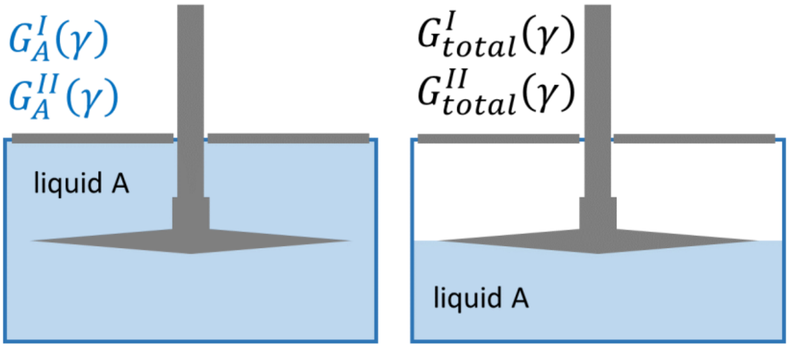


**Figure S6.** Measurement of surface viscoelastic properties - method based on calibration measurements: measurement with liquid A and at the surface.

If data are measured with sensor fully immersed in both liquids A and B and at the interface between A and B, then apparent elastic and viscous moduli are calculated according to Eqs. (S10), (S11) and (S12). This apparent elastic and viscous moduli of the bulk liquids A and B as a function of deformation $G_A^I(\gamma)$,

$G_A^{II}(\gamma)$, $G_B^{I}(\gamma)$, $G_B^{II}(\gamma)$, as well as the total elastic and viscous moduli corresponding to the contributions of two liquids and interface $G_{total}^{I}(\gamma)$, $G_{total}^{II}(\gamma)$ are available. Then the required information about the set of interfacial elastic and viscous moduli $G_i^{I}(\gamma)$, $G_i^{II}(\gamma)$ is given by subtraction of the individual bulk contributions, see Fig. S5:

$$G_i^I(\gamma) = G_{total}^I(\gamma) - \frac{G_A^I(\gamma)}{2} - \frac{G_B^I(\gamma)}{2}, \quad \text{(S13)}$$

$$G_i^{II}(\gamma) = G_{total}^{II}(\gamma) - \frac{G_A^{II}(\gamma)}{2} - \frac{G_B^{II}(\gamma)}{2}. \quad \text{(S14)}$$

When measuring at the liquid/air interface, see Fig. S6, these equations are reduced to:

$$G_i^I(\gamma) = G_{total}^I(\gamma) - \frac{G_A^I(\gamma)}{2}, \quad \text{(S15)}$$

$$G_i^{II}(\gamma) = G_{total}^{II}(\gamma) - \frac{G_A^{II}(\gamma)}{2}. \quad \text{(S16)}$$

The interface elastic and viscous moduli for DW-air, SS-air and MAH-air interfaces were calculated according Eqs. (S15) and (S16) while the interface elastic and viscous moduli for DW-SO, SS-SO and MAH-SO interfaces were calculated according Eqs. (S13) and (S14). It must be also mentioned that two types of oscillation measurements can be performed: (i) amplitude sweep (amplitude variation at constant frequency) or (ii) frequency sweep (frequency variation at constant amplitude). In this work, only amplitude sweeps are presented.

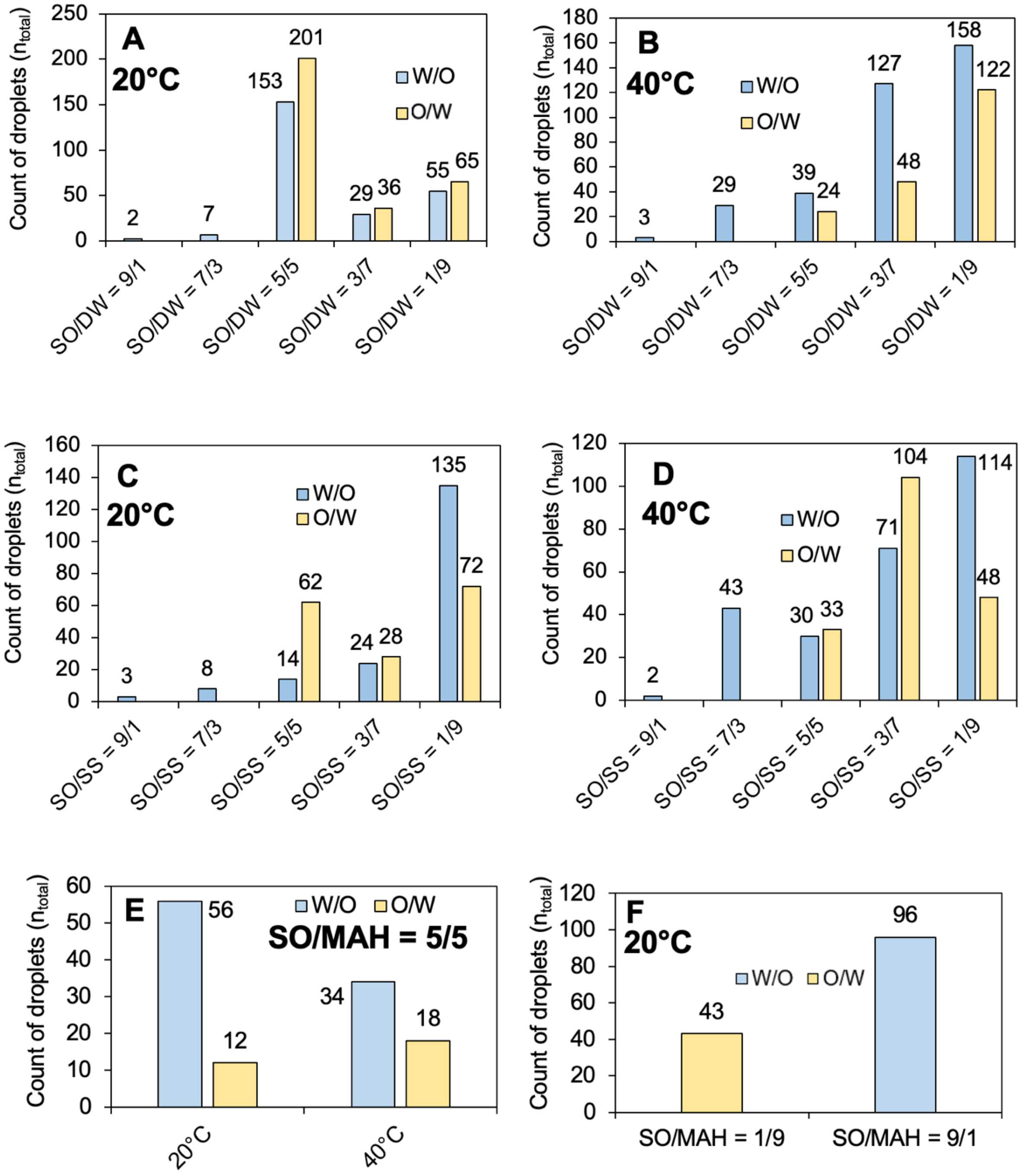


**Figure S7.** Total count of droplets in respective emulsions for experiments with distilled water (**A,B**), salt solution (**C,D**) and model aqueous solution (**E,F**).

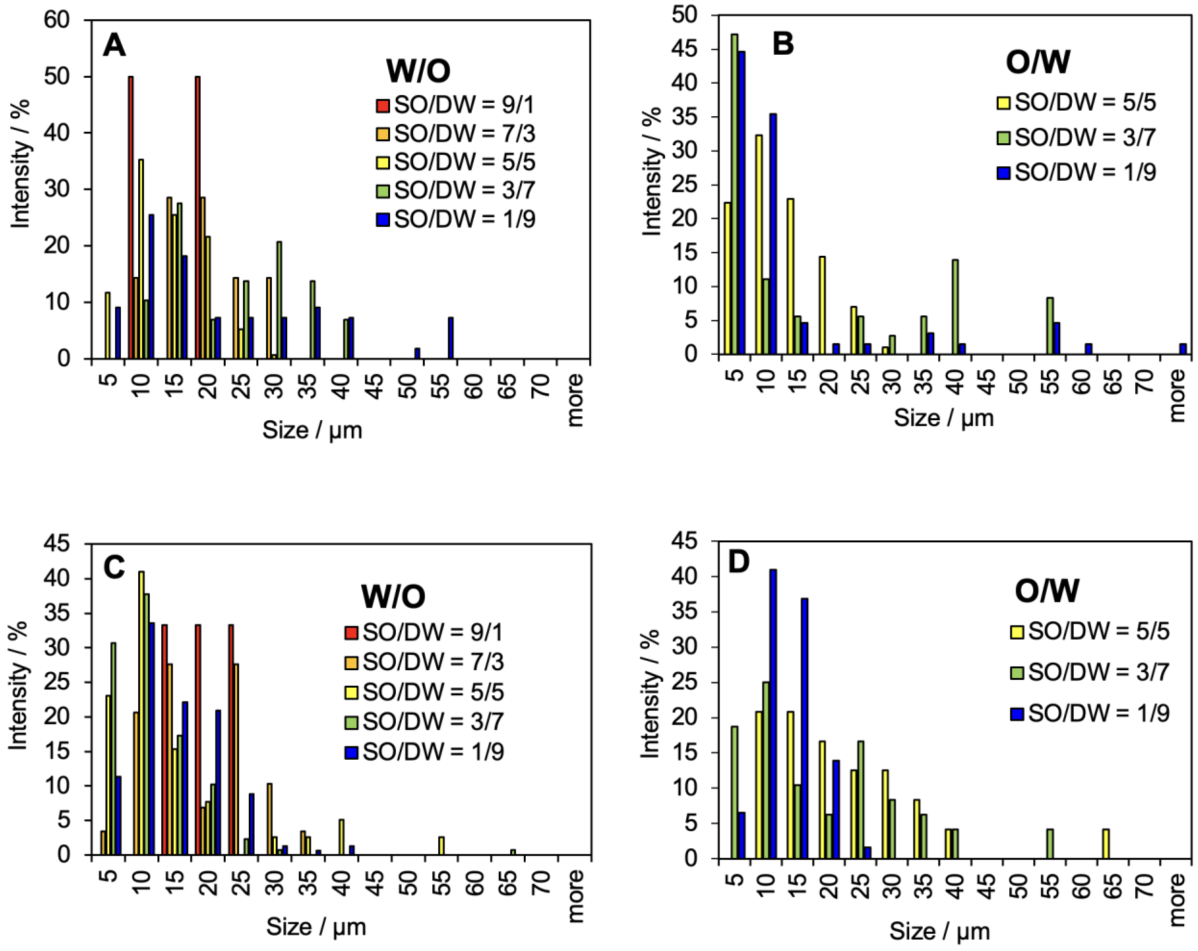


**Figure S8.** Droplets size distributions (in %) of emulsions obtained through SO emulsification experiments with distilled water (DW) at temperature 20 °C (**A,B**) and 40 °C (**C,D**).

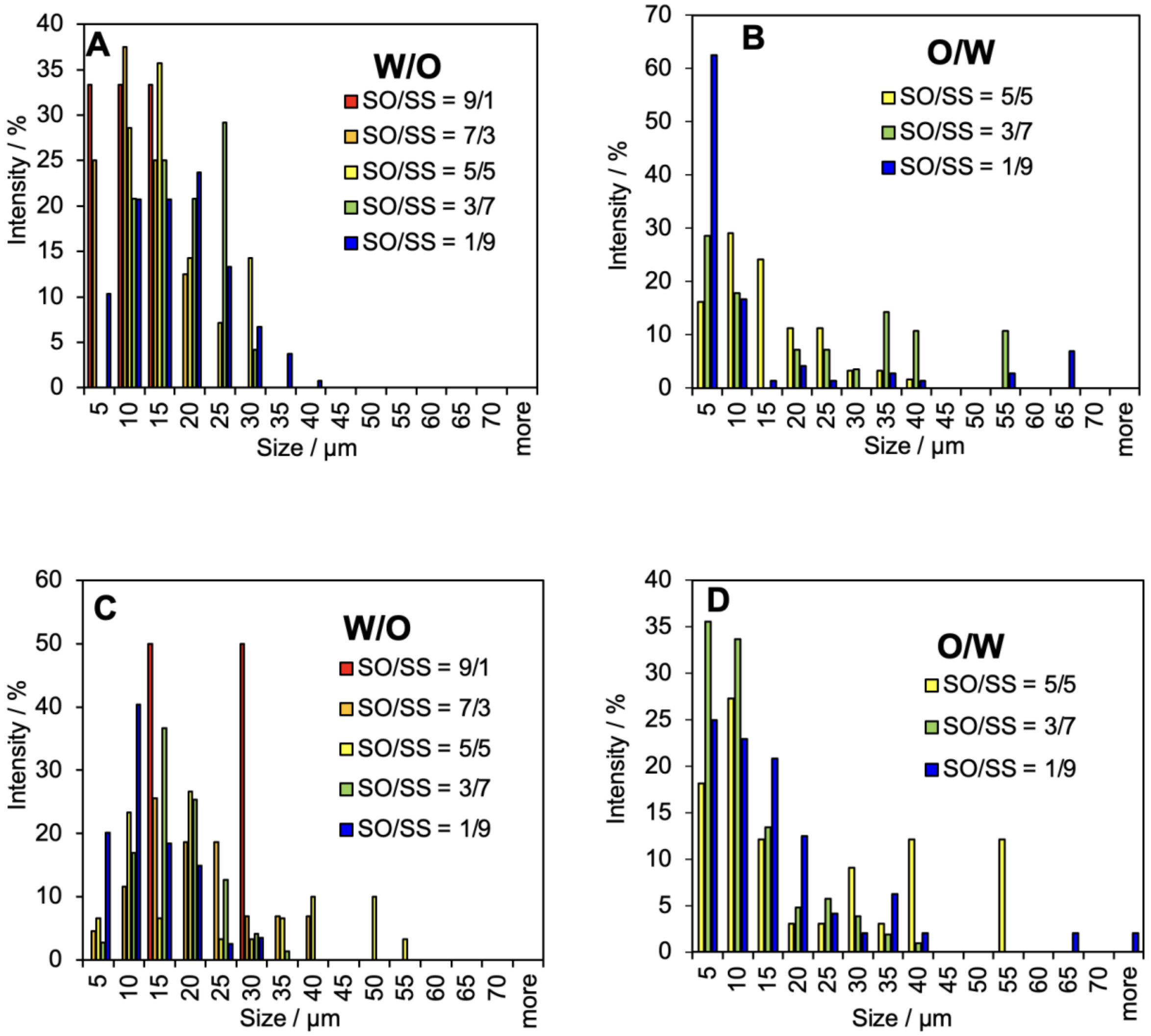


**Figure S9.** Droplets size distributions (in %) of emulsions obtained through SO emulsification experiments with salt solution (SS) at temperature 20 °C (**A,B**) and 40 °C (**C,D**).

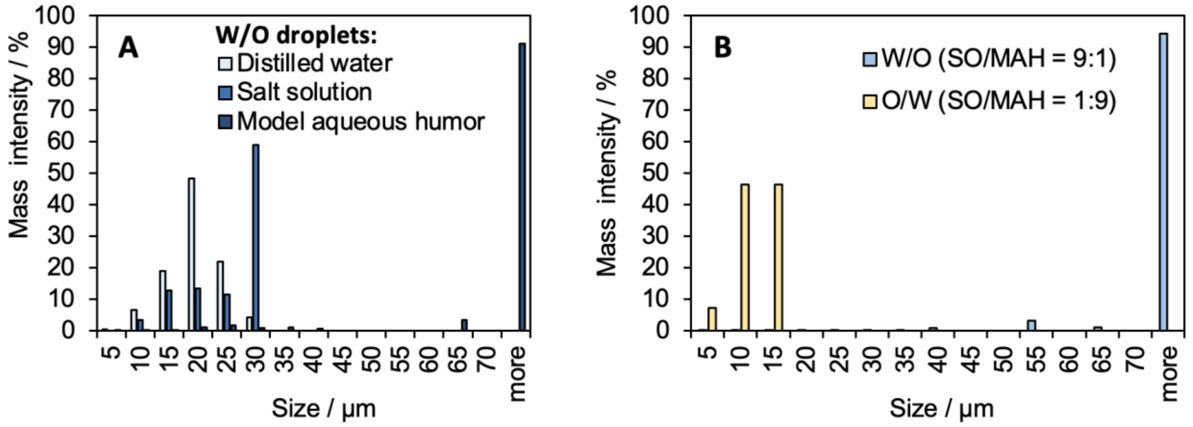


**Figure S10.** Comparison of water droplets mass distributions for DW, SS and MAH detected in emulsification experiments performed at oil/water ratio 5:5 (**A**) and for MAH detected in emulsification experiments performed at oil/water ratio 9:1 or 1:9 (**B**). *(All experiments at 20 °C)*

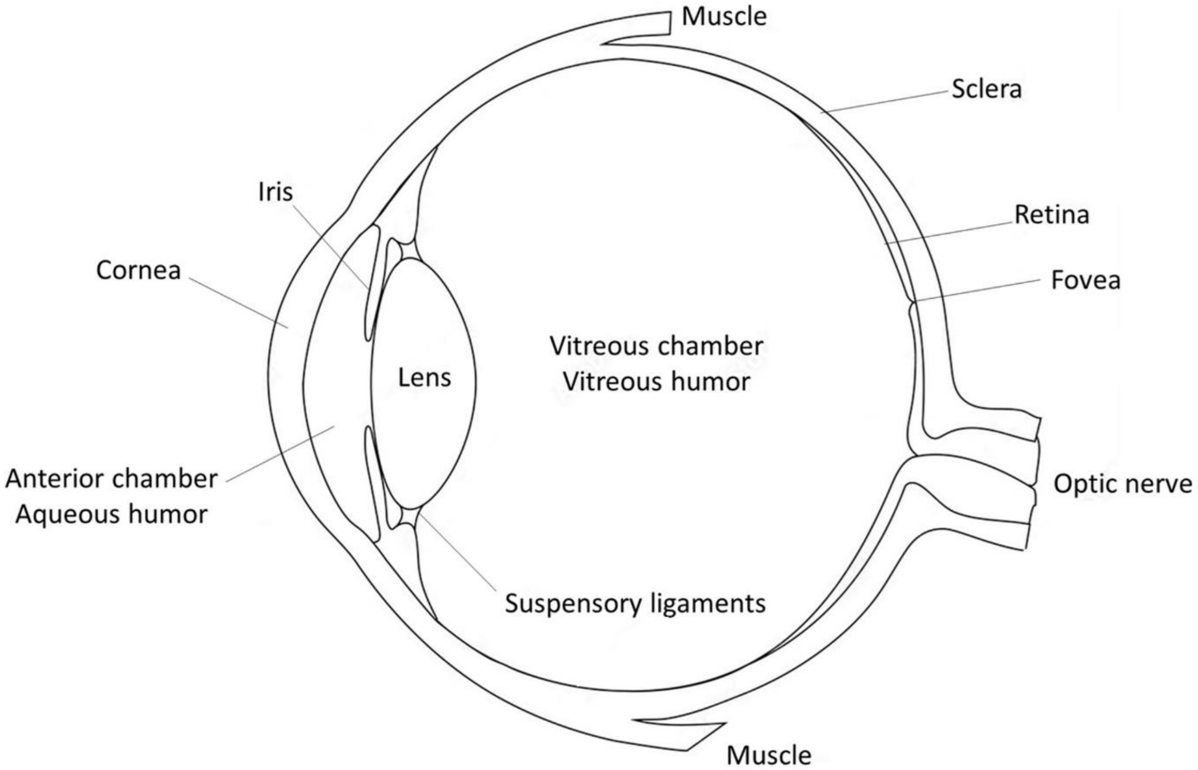


**Figure S11.** The structure of the eye.

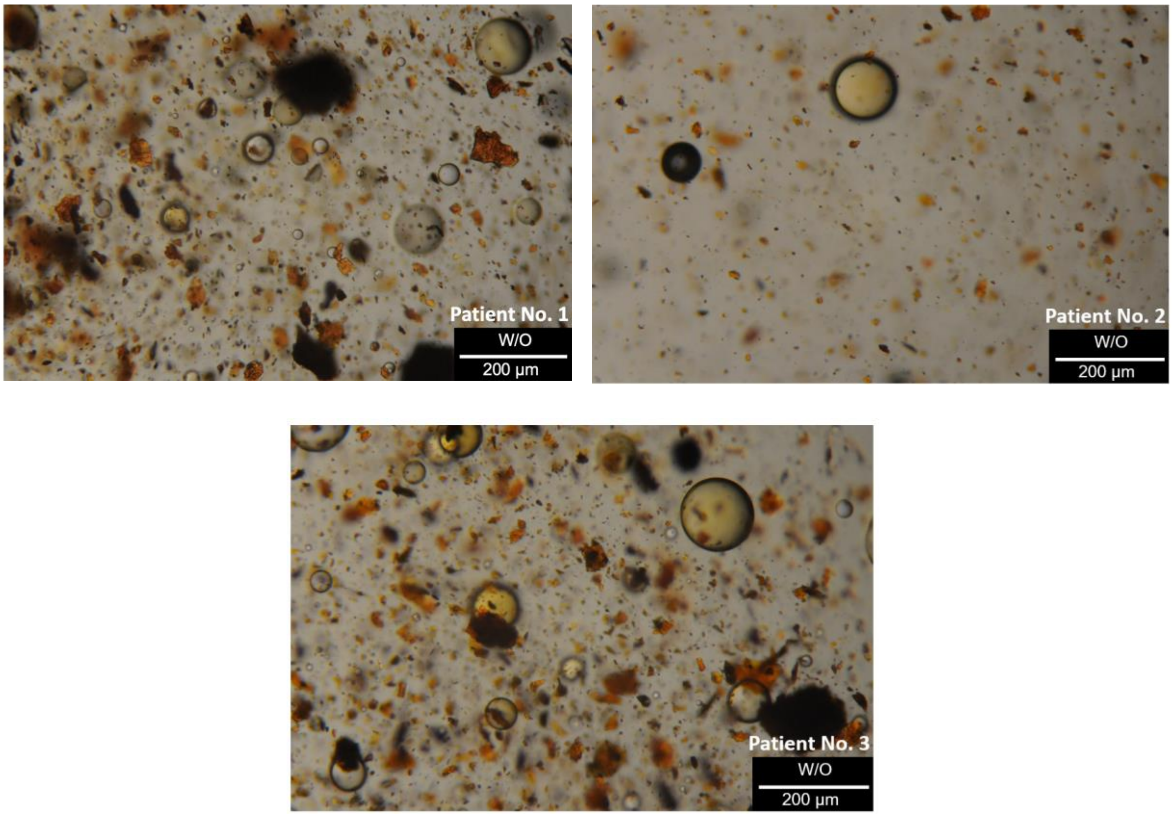


**Figure S12.** Examples of microscope images of the water droplets dispersed in medical-grade SO recovered from vitreous cavity. *The sample of medical-grade of SO removed from vitreous cavity after short-term tamponade from 3 patients was dyed with MO to confirm the presence of W/O droplets. The orange-colored background structures represent undissolved MO particles dispersed in the continuous oil phase. In the dyeing method, MO dissolves preferentially in the aqueous droplets of W/O emulsions, while remaining undissolved in the SO phase.)*

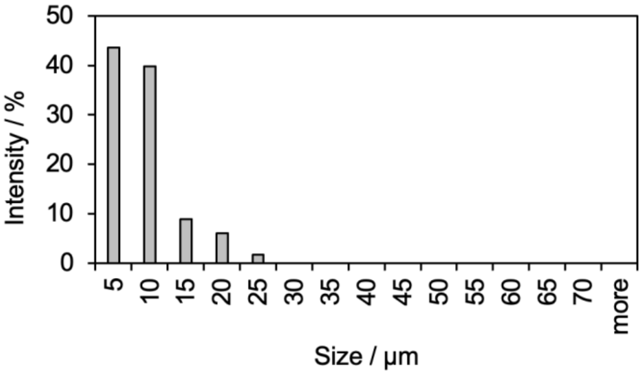


**Figure S13.** Droplets size distributions (in %) of emulsion obtained through vitreous humor emulsification experiments.

**Table S1** Results of emulsions stability.

| **Emulsion**[*] | **Aqueous phase** | **Stability** |
|---|---|---|
| W/O | Distilled water | 2 months |
| | Salt solution | 2 months |
| | Model aqueous humor | > 4 months |
| W/O | Distilled water | 2 months |
| | Salt solution | 2 months |
| | Model aqueous humor | > 4 months |

***Note*** – [*] *The oil/water phase ratio = 5:5; temperature 20 °C.*
